# Longitudinal Associations Between Depression Symptoms and Cognitive Functions in Chinese Older Adults: A Cross-Lagged Panel Network Analysis

**DOI:** 10.1155/da/3984020

**Published:** 2025-10-10

**Authors:** Hongfei Ma, Meng Zhao, Huimin Yin, Shuang Zhao, Pingmin Wei

**Affiliations:** ^1^Department of Epidemiology and Health Statistics, School of Public Health, Southeast University, Nanjing, Jiangsu, China; ^2^Hospital Office, The First Affiliated Hospital of Soochow University, Suzhou, Jiangsu, China

**Keywords:** cognitive function, cross-lagged panel network, depression symptom, longitudinal, older adults

## Abstract

**Background:**

With rapid population aging in China, understanding the relationship between depression symptoms and cognitive function is crucial for improving the mental health of older adults. This study investigates these dynamics using data from the China Health and Retirement Longitudinal Study (CHARLS).

**Methods:**

We analyzed data from the 2015, 2018, and 2020 waves of CHARLS, including 5203 participants aged 60 and above. Depression symptoms were measured using the Centre for Epidemiological Studies Depression-10 (CESD-10) scale, while cognitive function was assessed via the Mini-Mental State Examination (MMSE) scale. Cross-sectional network analysis was utilized for constructing the contemporaneous network, and cross-lagged panel network (CLPN) analysis was subsequently employed for longitudinal analysis.

**Results:**

In all three cross-sectional networks, “Hope” was identified as a key bridge symptom connecting the depression symptom community and the cognitive function community, while “Depressed mood” was found to be the central symptom of the entire network. In temporal networks, higher drawing ability at wave 1 was associated with greater “Hope” at wave 2, whereas higher “Fear” at wave 1 was related to lower recall ability at wave 2. Moreover, lower memory ability at wave 2 was associated with lower “Bothered” at wave 3.

**Conclusion:**

This study uncovered the dynamic interplay between specific depression symptoms and cognitive functions among Chinese older adults, thereby providing further validation for the scar theory and the cognitive vulnerability model. Additionally, it provides a critical theoretical foundation for developing intervention strategies targeting mental health and cognitive function in the aging population, as well as a scientific basis for related policy formulation. Future research should integrate quantitative and qualitative data for stronger causal validation.

## 1. Introduction

With the increasing global life expectancy of older adults, their duration of survival has extended, leading to a persistent rise in their proportion of the total population [1]. The phenomenon of population aging is equally severe in China. According to the results of the Seventh National Population Census, the population aged 60 and above in China is 264 million, accounting for 18.7% of the total national population [2]. Given the rapid pace of population aging, there is an urgent need to address the health challenges accompanying this demographic shift. Due to factors such as declining physical health, unstable economic income, and difficulties in daily life, older adults often exhibit more mental problems than younger people [3, 4]. Among the various mental problems faced by older adults, depression stands out as the most prevalent mental health concern. Depression is prevalent across all stages of life, with approximately one-third of older adults exhibiting depressive symptoms [5]. Furthermore, a previous study discovered that 13.3% of older adults globally reported major depressive disorder (MDD), and this prevalence is demonstrating an increasing trend within the global older adults [6]. As age advances, the course of depression worsens. Findings from a large cohort study revealed that, compared to younger adults, those aged 70 and above exhibited more severe symptoms and poorer prognoses [7]. Besides the common prevalence of depression among older adults, cognitive dysfunction also represents a significant concern in this population. Cognitive function in older adults involves multiple domains, encompassing memory, language, executive function, and attention, among others [8]. A meta-analysis including studies from 23 provinces in China revealed that the prevalence of mild cognitive impairment among older adults is 15.4% [9]. Unhealthy lifestyles, chronic diseases such as hypertension and diabetes, as well as psychiatric disorders like depression, have been identified as risk factors for cognitive impairment [9].

In China, the rapid aging of the population is accompanied by unique demographic, social, and cultural contexts that significantly impact the mental health and cognitive functioning of older adults. The traditional multigenerational family structure historically provided emotional support and daily care for older adults [10]. However, with the acceleration of urbanization and the reduction in family size, older adults in China are now facing the phenomenon of “empty nesting,” where adult children migrate for work, leaving older adults to live alone and at risk of social isolation [10, 11]. A study conducted in China found that social isolation is closely associated with depression and cognitive decline, particularly among older adults [12]. Moreover, the stigma surrounding mental health issues in Chinese society may lead to underdiagnosis and undertreatment of depression [13]. Many older adults may conceal their symptoms due to fear of being labeled as “mentally ill,” thereby delaying timely intervention [13]. These factors collectively contribute to the unique complexity and importance of studying the relationship between depression and cognitive functioning in the Chinese context.

Compared to younger individuals, the onset and progression of depression and its symptoms in older adults are more closely associated with various functional and cognitive impairments [5]. This relationship is consistent with the scar theory model, which posits that depression can temporarily or permanently impair an individual's cognitive function [14]. Even if depression symptoms are no longer apparent in the future, these scars may persist, continuing to affect cognitive function [14]. The impact of depression on cognitive functioning in older adults can be integrated from three perspectives: biological, psychological, and social. From a biological perspective, depression may be associated with hypothalamic axis dysregulation, neurotransmitter imbalances, and changes in brain structure, all of which can directly impact cognitive functions, particularly working memory, processing speed, and verbal memory [15, 16]. From a psychological perspective, the lifespan model of inhibitory control has established that depressive mood serves as a pivotal risk factor for future decline in executive function [4]. From a social perspective, older adults with depression often perceive themselves to be isolated and lonely [17]. Depression symptoms frequently hinder individuals from engaging in normal social activities, impairing their social functioning, which, in turn, can lead to cognitive decline or impairment [14]. A previous study has also examined the long-term impact of depression on cognitive dysfunction in older adults, further supporting the scar theory. A longitudinal study on the physical and mental health of older adults in China found that different states of depression symptoms are associated with cognitive function, with participants exhibiting persistent depression symptoms experiencing a more rapid decline in cognitive function [18].

The scar theory provides a framework for understanding the impact of depression on cognitive function, while the cognitive vulnerability model further reveals how cognitive decline can deeply influence depression. The basic assumption of the cognitive vulnerability model posits that individuals with depression tend to have a negative and fragile self-perception, considering themselves worthless [14]. This negative perception is often understood as one of the risk factors leading to depression symptoms. Recent studies have revealed that a decline in cognitive functions is likely to lead to future depressive somatic/autonomic symptoms, diminished positive affect, and increased interpersonal issues [15, 19]. The association between cognitive decline and depression and other mental health disorders is particularly pronounced in older adults. A meta-analysis, encompassing 13 cross-sectional and 4 prospective studies, revealed that in older adults, cognitive impairment related to dementia can potentially serve as a risk factor for depression [20]. Compared to younger and middle-aged individuals, the relationship between executive function and depression symptoms in older adults is notably pronounced, with deficits in executive functioning potentially leading to depression [21]. Furthermore, understanding the bidirectional relationship between cognitive function and depression is crucial, as cognitive decline can exacerbate depression symptoms, while depression can further impair cognitive abilities, creating a complex interplay that significantly affects individuals. Some longitudinal studies and meta-analyses have demonstrated a potential bidirectional causal relationship between depression symptoms and cognitive function, wherein cognitive decline and the exacerbation of depression symptoms mutually influence each other [4, 22, 23]. This interdependent relationship is particularly alarming in older adults, as cognitive impairment and depression symptoms frequently manifest concurrently with advancing age.

However, existing literature has predominantly focused on the general relationship between depression and cognitive function, often adopting a holistic approach by constructing models based on overall scores from depression and cognitive function scales, but it lacks an in-depth exploration of specific symptoms and their interactions over time [22]. The recently developed network analysis approach allows for quantifying the relationships between symptoms in psychiatric disorders, identifying closely related symptom clusters while also detecting central symptoms that sustain the network [8, 24, 25]. Some researchers have endeavored to employ cross-sectional network analysis methods to uncover bridge and central symptoms that exhibit strong associations between cognitive function and depression symptoms within particular demographics. For instance, a cross-sectional network analysis examining cognitive function and depression symptoms in older adults revealed that language ability, naming ability, and “difficulty with concentrating” were identified as transdiagnostic bridge symptoms [8].

While cross-sectional network analyses provide valuable insights into the associations between cognitive function and depression symptoms at a single point in time, they fail to capture the dynamic, temporal, and causal relationships between these symptoms [26]. Cross-lagged panel network (CLPN) analysis overcomes these limitations by allowing researchers to investigate how these relationships evolve across multiple time points, revealing temporal and causal pathways [26–28]. In previous studies, researchers have employed CLPN to investigate the longitudinal associations and development of psychological health symptoms among adolescents [27, 29, 30], middle-aged individuals [15], and other special populations [31]. Despite evidence indicating that the cognitive-mental health network relationships may be more pronounced in older adults compared to younger and middle-aged individuals [4], research utilizing CLPN to explore the longitudinal relationships between specific depression symptoms and cognitive functions in Chinese older adults remains limited. Schlechter et al. [32] conducted a CLPN analysis on depression symptoms among 11,391 older adults in the English Longitudinal Study of Ageing. However, this study focused solely on the longitudinal relationships between internal depression symptoms and did not examine their associations with cognitive functions. Another longitudinal study analyzed nine psychopathological nodes and eight cognitive function nodes in older adults, finding that anxiety and depression had the strongest associations with executive function impairment measured approximately 2 years later [4]. Nevertheless, this study did not specifically examine the relationships between individual depression symptom nodes and cognitive function nodes; instead, it treated depression as a single node in constructing the longitudinal network.

This study aims to explore the longitudinal relationships between specific depression symptoms and cognitive functions among Chinese older adults using CLPN analysis. Previous research has utilized network analysis to uncover connections between individual disease symptoms, but the application of longitudinal network analysis, particularly in examining the dynamic and temporal relationships between specific depression symptoms and cognitive functions, remains underexplored, especially in the context of older adults. This gap highlights the need for a more detailed investigation into how these relationships evolve over time, providing a foundation for the objectives of this study.

## 2. Methods

### 2.1. Participants and Procedures

The data for this study were collected from the China Health and Retirement Longitudinal Study (CHARLS) [33]. CHARLS is a nationally representative longitudinal survey conducted in China among individuals aged 45 and above, encompassing assessments of participants' social, economic, physical, and mental health status [32]. This project has been tracked every 2 or 3 years, and there are currently five waves of data: 2011, 2013, 2015, 2018, and 2020 [34]. The data collection for CHARLS employed the probability proportional to size (PPS) sampling method, which ensured the representativeness of the selected samples [34].

We selected the data from the last three waves of the CHARLS survey for our study, as they most comprehensively encompass the cognitive function and depression symptoms we aim to investigate. Our study exclusively includes participants who have engaged in all three rounds of the survey (*N* = 16,971), which enhances the internal consistency of the data and mitigates the issues of sample variability arising from changes in participants across different rounds. After excluding participants who were under 60 years of age at the time of the 2015 survey (65 years and under at the time of the 2020 survey) and those who refused to answer all questions related to cognitive function or depression in any wave, the final sample for this study comprised 5203 individuals. The specific participant selection process is shown in Figure S1. The CHARLS survey obtained ethical clearance from Peking University's Ethical Review Committee (Approval Number IRB00001052-11015), and all participants signed an informed consent form in writing before participating in the three rounds of the research study [35].

### 2.2. Measurements

This study used the Centre for Epidemiological Studies Depression-10 (CESD-10) to assess depression symptoms in older adults. The CESD-10 is a self-report questionnaire consisting of 10 items, each corresponding to a different depression symptom [36]. Scores range from 0 to 30, with higher scores indicating more severe depression symptoms. To date, the CESD-10 has been extensively utilized in research involving Chinese populations, and the tool has been demonstrated to possess robust reliability and validity [36, 37]. In this study, Cronbach's *α* for the CESD-10 across the first, second, and third waves of data collection was found to exhibit high internal consistency, with values of 0.793, 0.789, and 0.783, respectively.

Although the questions assessing cognitive function in the three waves of the CHARLS project were not entirely consistent, they were all built upon the foundation of the Mini-Mental State Examination (MMSE), with variations in the number of items added or removed across different waves. In our study, we extracted the commonly measured cognitive functions from the three rounds of surveys, which include orientation ability, memory ability, attention, recall ability, and drawing ability. Higher scores on the cognitive functioning component indicate better cognitive functioning. Previous research has demonstrated that the MMSE scale has been employed in network analysis among older adults in China and exhibits high reliability [8, 36]. The internal consistency of the three rounds of cognitive function survey questions was generally high, with Cronbach's *α* coefficients ranging from 0.733 to 0.766.

### 2.3. Statistical Analyses

In our study, all statistical analyses were performed using R (Version 4.3.3). Prior to conducting the network analysis, multiple imputation for missing values in cognitive function and depression symptoms was performed using the mice R package [38]. Multiple imputation was a gold standard approach for data that is likely missing at random and maximizes power by using all relevant available data [4, 38]. Multiple imputation offered significant advantages in longitudinal studies. Longitudinal datasets often had missing data due to participant dropout, nonresponse, or other factors. Multiple imputation generated multiple plausible imputations to fill in the missing data, thereby preserving sample size and statistical power [39]. Unlike complete case analysis or single imputation methods, multiple imputation accounted for the uncertainty of missing data by generating multiple datasets and pooling the results, thereby reducing bias and providing more accurate estimates of parameters and their variances [40]. Moreover, the use of multiple imputation for handling missing data was widely applied in studies of symptom network analysis and was shown to minimize bias and improve efficiency [4, 15]. This method was particularly suitable for regularized regression analysis as compared to nonregularized regression analysis [4].

After completing the imputation of missing values, we constructed contemporaneous networks for three waves using the qgraph R package [41, 42]. The qgraph R package was particularly well-suited for constructing contemporaneous networks, which represented relationships between variables at a single time point. Specifically, it implemented the Extended Bayesian Information Criterion (EBIC) and the graphical least absolute shrinkage and selection operator (LASSO) network model, which were essential for constructing sparse and interpretable contemporaneous networks [41, 42]. These methods regularized partial correlations, reduced the risk of overfitting, and ensured that only meaningful connections were retained in the network. Beyond its robust modeling capabilities, the qgraph R package also provided intuitive visualizations of network structures, where nodes represented specific depression symptoms and cognitive functions, and edges represented partial correlations between them. The width of the edges indicated the strength of the correlations, while the colors (blue for positive correlations and red for negative correlations) denoted the direction of the relationships. We investigated the central symptoms in the contemporaneous network by utilizing strength and expected influence (EI). Moreover, we used bridge strength (BS) and bridge expected impact (BEI) to assess the symptom connections across communities.

To investigate the longitudinal causal relationships between cognitive functions and depression symptoms across three different waves, we constructed two CLPN models using the glmnet R package [43]. The glmnet R package was primarily used to construct longitudinal relationships between variables, including the autoregressive and cross-lagged relationships in the CLPN by utilizing the LASSO regularization technique [43, 44]. Specifically, autoregressive relationships refer to the prediction and influence coefficients of a symptom node at wave 1 on the same node' s symptoms at wave 2 (controlling for other symptom nodes at wave 1) [45]. Cross-lagged regression relationships refer to the prediction and influence coefficients of a symptom node at wave 1 on different symptoms at wave 2 (controlling for other symptom nodes at wave 1) [45]. In this study, the LASSO regularization technique implemented in the glmnet R package, combined with applied 10-fold cross-validation, helped mitigate the influence of spurious edges by shrinking weak or irrelevant coefficients to zero. This was particularly crucial in longitudinal network analysis, as the goal was to identify meaningful causal relationships while avoiding excessive irrelevant connections. In the temporal network, the color of the arrows denotes the direction of the effects, with blue arrows indicating positive effects and red arrows indicating negative effects. The thickness and intensity of the lines represent the strength of the associations. For the directed cross-lagged network, we computed the in-prediction (predictability) and out-prediction (influence) values for each symptom node across the two temporal networks. The predictability value of a node reflects the extent to which the node in wave 2 is influenced by all other nodes from wave 1 [30]. In contrast, the influence value of a node represents the degree to which the symptoms of that node in wave 1 affect the symptoms of all other nodes in wave 2 [30].

The accuracy and stability of the contemporaneous and temporal networks were examined using the bootnet R package [31, 41]. First, to assess the precision of the edge weights, we applied 95% confidence intervals around each weight using nonparametric bootstrapping, conducted over 5000 iterations [31, 41]. Second, network stability was examined by the case-dropping subset bootstrap function (5000 iterations) with a correlation stability coefficient (CS coefficient) [31, 41]. A CS coefficient exceeding 0.50 indicates high stability and strong interpretability. Finally, we conducted significance tests for edge weights and centrality differences in both contemporaneous and temporal networks to explore whether the edge weights and centrality indices significantly differ from one another [41].

## 3. Results

### 3.1. Descriptive Statistics

The basic demographic characteristics of the 5203 older adults who participated in all three waves of the survey are presented in Table 1. The gender distribution among the 5203 participants showed a small difference (48.91% male vs. 51.09% female), with the highest number of individuals falling within the age range of 65–69 years (46.38%). In terms of marital status, the majority of respondents were married (70.52%), and most participants lived in rural areas (70.61%).  Table 2 presents the means and standard deviations for all items of cognitive function and depression symptoms across the three waves of surveys, as well as the skewness and kurtosis for each symptom. Over the three waves, cognitive function showed variation, with recall improving in wave 3, while orientation and memory declined slightly across the waves. Depression symptoms remained relatively stable, though there were slight increases in certain items like mind distraction and exhaust.

### 3.2. Contemporaneous Networks

**Figure 1:**
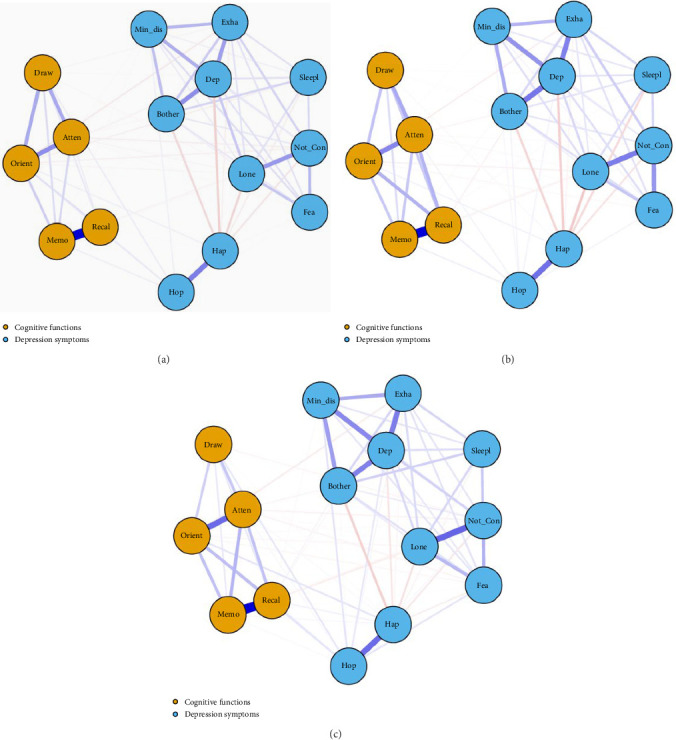
Contemporaneous networks of cognitive functioning and depression components: (a) shows the wave 1 (2015), (b) shows the wave 2 (2018), and (c) shows the wave 3 (2020). Each node represents an individual symptom of the participant in the analysis. Edges represent the relationship between two nodes after conditioning on all other nodes in the analysis. Positive associations are denoted by blue edges, while negative associations are represented by red edges. The thickness of the lines indicates the strength of the associations. Yellow nodes represent cognitive functions, and blue nodes represent depression symptoms. The abbreviations and full names of each function or symptom are as follows: Atten, attention; Bother, bothered; Dep, depressed mood; Draw, drawing; Exha, exhaust; Fea, fear; Hap, happy; Hop, hope; Lone, loneliness; Memo, memory; Min_dis, mind distraction; Not_Con, cannot continue; Orient, orientation; Recal, recall; Sleepl, sleeplessness.

The cognitive function and depression contemporaneous networks across the three waves are illustrated in Figure 1. Within the cognitive function community, the results from three waves of contemporaneous network analyses consistently revealed that the relationship between “Memory” and “Recall” was the closest (*r*_wave 1_ = 0.645, *r*_wave 2_ = 0.546, *r*_wave 3_ = 0.532). Within the depression community, the strongest edges identified in the first two waves of network analyses were between “Hope” and “Happy” (*r*_wave 1_ = 0.321, *r*_wave 2_ = 0.307), while in the wave 3 network, the most prominent edge was between “Loneliness” and “Cannot continue” (*r*_wave 3_ = 0.324). In the cross-diagnostic contemporaneous networks, the most closely connected edges across communities in the wave 1 and wave 3 networks were between “Orientation” and “Hope” (*r*_wave 1_ = 0.046, *r*_wave 3_ = 0.053). In the wave 2 network, the most closely connected edge across communities was between “Memory” and “Hope” (*r*_wave 2_ = 0.036). In the analysis of the three-wave network, the correlation matrices between other symptomatic nodes can be found in Tables S1–S3. All three waves of contemporaneous network analysis results indicated that “Depressed mood” was the central symptom across the network (EI_wave 1−3_ = 1.18–1.76), with the centrality of other symptoms presented in Table S4 and Figure S2. “Hope” exhibited the highest BEI among all symptoms, serving as a bridging centrality symptom between the cognitive function and depression communities (refer to Figure S3).

### 3.3. Temporal Networks

**Figure 2:**
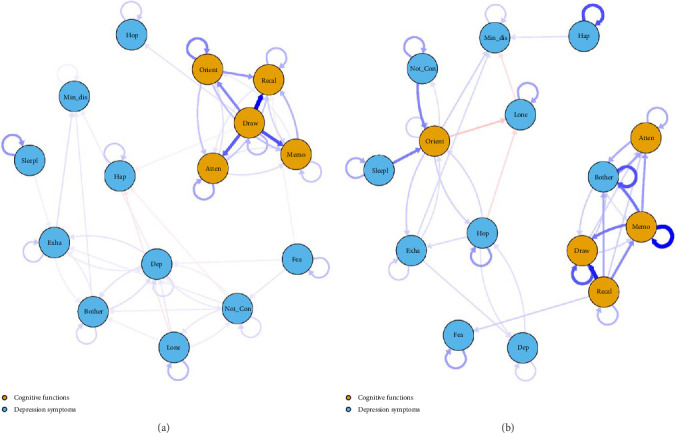
The cross-lagged panel networks for wave 1 → wave 2 (a) and wave 2 → wave 3 (b). Yellow nodes indicate cognitive function, and blue nodes indicate depression symptoms. Arrows represent unique longitudinal relationships. Between different nodes, blue edges indicate positive relationships and red edges indicate negative relationships. The circular arrow linking to itself represents autoregressive influence. The thickness and color depth of the edge represent the strength of the relationship; the thicker the edge, the stronger the relationship. The abbreviations and full names of each function or symptom are as follows: Atten, attention; Bother, bothered; Dep, depressed mood; Draw, drawing; Exha, exhaust; Fea, fear; Hap, happy; Hop, hope; Lone, loneliness; Memo, memory; Min_dis, mind distraction; Not_Con, cannot continue; Orient, orientation; Recal, recall; Sleepl, sleeplessness.

The cross-lagged network results (wave 1→wave 2) for cognitive function and depression are shown in Figure 2a. “Orientation” (*β* = 0.380), “Sleeplessness” (*β* = 0.326), and “Attention” (*β* = 0.285) were the nodes with the greatest autoregression coefficients (refer to Figure S8). In the cross-lagged network model, higher drawing ability at wave 1 was associated with greater recall ability (*β* = 0.784) and memory ability (*β* = 0.541) at wave 2. In terms of cross-diagnostic influences, higher drawing ability at wave 1 was associated with greater “Hope” at wave 2 (*β* = 0.083). This finding suggests that improvements in drawing ability among older adults may contribute to increasing their levels of hope over time. Conversely, higher “Fear” at wave 1 was related to lower recall ability at wave 2 (*β* = −0.056). This result indicates that elevated levels of fear may negatively impact recall ability over time. Additional cross-lagged relationships from wave 1 to wave 2 are shown in Table S5.

The cross-lagged network results (wave 2→wave 3) for cognitive function and depression are shown in Figure 2b. “Memory” (*β* = 0.460), “Happy” (*β* = 0.319), and “Bothered” (*β* = 0.316) were the nodes with the greatest auto-regression coefficients (refer to Figure S8). In the entire network, the influence of recall ability in wave 2 on drawing ability in wave 3 was the strongest, with higher levels of recall ability being associated with higher levels of drawing ability (*β* = 0.409). In terms of cross-diagnostic influences, lower memory ability at wave 2 was associated with lower “Bothered” at wave 3 (*β* = 0.254). This result implies that declines in memory ability may lead to reduced feelings of being bothered over time. Additional cross-lagged relationships from wave 2 to wave 3 are shown in Table S6.

**Figure 3:**
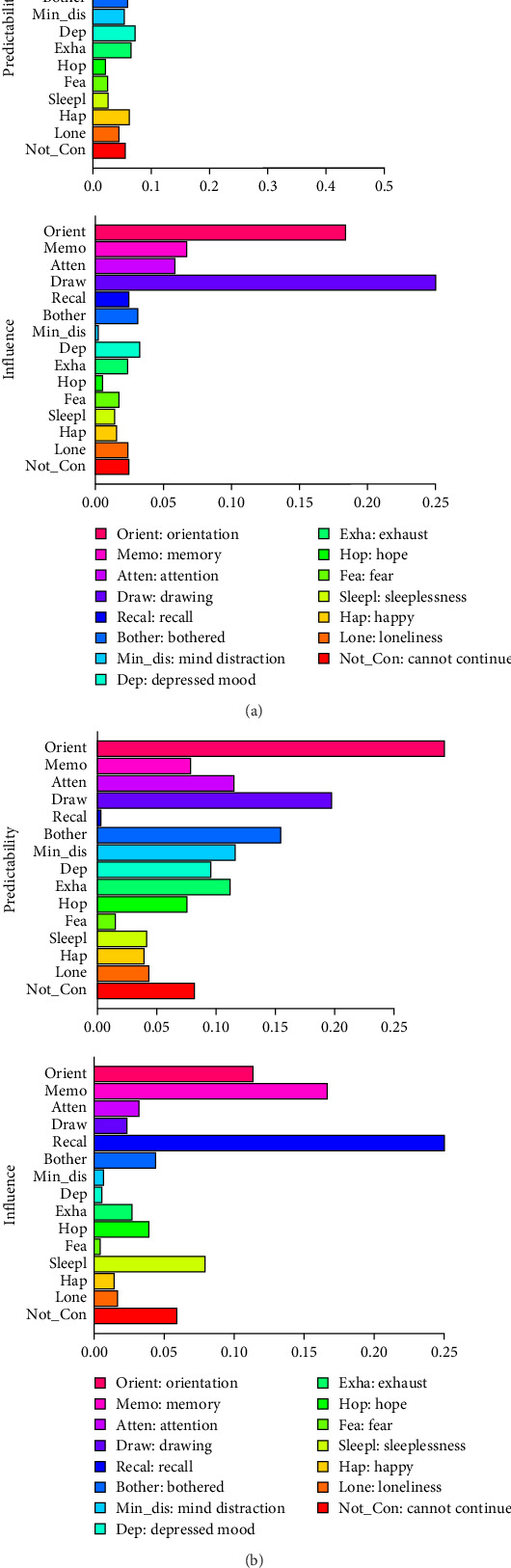
The cross-lagged in-prediction (predictability) and out-prediction (influence) in the estimated temporal network: (a) represents the result of wave 1 to wave 2 and (b) represents the result of wave 2 to wave 3.

Figure 3a shows the cross-lagged centrality results from wave 1 to wave 2 phases. Recall ability and memory ability were not drivers in the network, given very high in-prediction values but very low out-prediction values. Instead, the most influential node with low in-prediction and high out-prediction values was drawing ability. Figure 3b shows the cross-lagged centrality results from wave 2 to wave 3 phases. Based on the characteristics of high in-prediction and low out-prediction for orientation abilities at this stage, this node does not serve as a driving factor within the network. In contrast, symptoms characterized by low in-prediction and high out-prediction capabilities included recall ability, which served as a significant influencing factor within the cross-lagged network. Specific values for the in-prediction and out-prediction metrics of other symptoms are presented in Table S7.

### 3.4. Network Accuracy and Stability

The CS coefficients for both the contemporaneous and temporal networks in this study exceeded 0.5, indicating that the centrality metrics of all nodes in these networks demonstrated high stability (refer to Figures S5 and S10). It is important to note that the CS coefficient does not provide node-specific stability metrics, as it is calculated for the entire network rather than individual nodes. Figures S4 and S9 demonstrated that there was a considerable overlap between the 95% confidence intervals of the edge weights, indicating that the edges of the network were trustworthy. Furthermore, results of the nonparametric bootstrap procedure indicated that most comparisons among edge weights and centrality indicators were statistically significant (refer to Figures S6 and S7).

## 4. Discussion

Under the frameworks of cognitive vulnerability theory and scar theory, cognitive function and depression symptoms are understood to interact with each other, with this interaction potentially strengthening as individuals age. However, there is limited research on the mechanisms of interaction between specific cognitive functions and specific symptoms of depression in older adults. This study aimed to explore the complex relationship between depression symptoms and cognitive functions in older adults through CLPN analysis, providing a systematic perspective. Our analysis of temporal networks revealed significant insights into how these domains interact over time. We present credible explanations for our research findings in order to further develop and refine the theoretical understanding of this study.

In the contemporaneous transdiagnostic network, “Hope” proved to be the most important bridging symptom between cognitive function and depression symptom clusters. This finding is consistent with the tenets of positive psychology, which suggest that positive psychological factors such as social support, optimism, and hope can enhance cognitive function and reduce the occurrence of negative emotions, thereby aiding in the resistance to various diseases [46]. Importantly, this observation provides a preliminary understanding of the “scar theory,” which suggests that cognitive decline and emotional symptoms may mutually reinforce each other over time [14]. While our findings from the contemporaneous network analysis do not directly capture the dynamic, mutual reinforcement described by the “scar theory,” they suggest that hope may act as a protective factor, potentially mitigating the negative feedback loop between cognitive decline and emotional symptoms. A clinical trial conducted among older adults confirmed the relationship between hope and depression symptoms, indicating that hope-based training can effectively alleviate depression symptoms in this cohort [47]. This underscores the potential of hope as a key intervention target in breaking the cognitive-emotional interplay described in the “scar theory.” Clinicians can enhance hope levels in older adults through cognitive-behavioral therapy and life review-based interventions [48], thereby further improving depression and cognitive function in this population. Additionally, art therapy, which has been demonstrated to enhance emotional expression and cognitive engagement, can serve as an effective adjunct to promote hope and improve cognitive outcomes [49, 50]. Based on our findings from the contemporaneous network analysis, interventions should focus on the hope-orientation and hope-memory relationships to weaken the associations within the cognitive function-depression symptom network.

The results of the contemporaneous network analyses across the three waves consistently identify “depressed mood” as the central symptom within the network. In symptom network theory, central symptoms are believed to activate or exacerbate the development of other symptoms within the network [37]. Depressive mood may promote or exacerbate the development of other related symptoms through a variety of mechanisms. On the one hand, depressive mood can disrupt emotional regulation, making it difficult for individuals to manage stress and anxiety effectively, thus creating a feedback loop that reinforces other depression symptoms. On the other hand, depressive mood is closely related to cognitive function, with research indicating that it is a critical risk factor for future decline in executive function [4]. Furthermore, social withdrawal often accompanies a depressed mood, reducing social support and increasing feelings of isolation, which can further deepen depression symptoms [14, 17]. The result that depressive mood serves as a central symptom is not only reflected in previous network studies of older adults but also validated in other specific disease groups [8, 51]. The consistency of these findings suggests that targeting depressive mood could be an important therapeutic or policy intervention goal. Current research has found that mindfulness-based cognitive therapy can improve depressive mood in older adults [52]. Therefore, by implementing this intervention, clinicians may not only help alleviate depression symptoms among older adults but also improve other cognitive functions within the network structure.

In the CLPN analysis, higher drawing ability in wave 1 was found to be associated with higher “hope” in wave 2. This finding can be interpreted through the association between art therapy and positive psychology [49]. Specifically, art therapy reconstructs the connections between cognition, emotion, and sensory experiences, which can enhance and develop healthy coping abilities and positive psychological factors to resist stress [49]. Drawing ability, as a form of artistic expression, bears a significant and close association with the mental health of individuals. A bibliometric analysis of studies on painting therapy indicates a sustained upward trend in research in recent years, highlighting its significant role in improving mental and physical health [53]. Previous studies have demonstrated that drawing interventions can significantly enhance participants' positive emotions [54], particularly by increasing their understanding of a sense of hope to stimulate their resilience [50]. In future intervention programs targeting depression symptoms among older adults, enhancing drawing ability should be considered an important strategy. At the same time, we found that higher “Fear” at wave 1 was related to lower recall ability at wave 2. This finding is consistent with the theories regarding the effects of fear and stress. The persistent sense of fear can trigger a stress response, and excessive stress may impair the function of brain regions associated with memory and recall, thereby weakening an individual's ability to retrieve and recollect information [55]. In addition, according to the Attentional Control Theory, anxiety (a manifestation of fear) can undermine the efficient functioning of the goal-directed attentional system and increase susceptibility to the influence of the stimulus-driven attentional system [56]. This implies that when individuals experience fear, they may allocate more cognitive resources to attend to threat-related stimuli, thereby reducing the cognitive resources available for other tasks such as recall and memory.

Interestingly, our CLPN analysis revealed an association between lower memory ability in wave 2 and decreased “Bothered” levels in wave 3. However, this may be inconsistent with the findings of previous studies that identified a negative correlation between cognitive function and depression symptoms [8, 15]. The observed differences in results may be due to temporal effects, as the relationship between specific cognitive functions and nodes of depression symptoms may change with aging in older adults. As individuals age, there is a general decline in memory among older adults, with deterioration becoming particularly pronounced after the age of 62 [57]. Previous research suggested that memory decline might be due to the selective forgetting of negative memories, which could, in turn, foster positive emotions [58]. This phenomenon is more prevalent among older adults compared to younger individuals [59]. As their perception of time changes, older adults tend to focus more on positive emotional experiences, which may consequently reduce how much they are bothered [59]. Nevertheless, the mechanisms underlying the positive association between memory ability and levels of being bothered are not yet fully elucidated, necessitating further studies to validate this relationship in older adults.

In the analysis of two temporal networks, it was observed that during the transition from wave 1 to wave 2, recall ability was most susceptible to the influence of other symptoms, consistent with previous findings that highlight the vulnerability of memory in early stages of cognitive decline [60, 61]. However, from wave 2 to wave 3, recall ability emerged as a significant factor impacting other cognitive functions and depression symptoms. This dual-phase dynamic highlights the evolving role of recall ability in the cognitive-depression network, transitioning from a state of high receptivity to one of significant influence. This is a unique finding in our study of the dynamic relationship between cognitive function and depression symptoms among Chinese older adults. In older adults, early cognitive decline makes memory recall susceptible to other factors, such as anxiety, depression, or other cognitive impairments, which can further affect the retrieval and preservation of memory [60, 61]. Over time, accumulated memories and experiences gradually give the ability to recall the power to influence other cognitive processes and emotional responses. Older individuals may progressively develop more complex memory strategies and cognitive frameworks, potentially leading to recall ability having a more significant influence on other cognitive functions and depression symptoms. This novel finding addresses the limitations of previous studies in Chinese older adults, which only conducted cross-sectional network analyses of the relationship between depression symptoms and cognitive function [8, 62]. The results of the dual-phase dynamic changes underscore the importance of targeted interventions in older adults during stages where recall ability is significantly impacted, potentially enhancing subsequent positive effects on cognitive function and emotional health.

Considering that the study population is Chinese older adults, Chinese cultural values may influence the relationship between depression symptoms and cognitive functioning found in this study. For instance, the strong cross-lagged relationship between higher drawing ability at wave 1 and greater “Hope” at wave 2 may be influenced by family support and intergenerational relationships. Positive family interactions can enhance cognitive functioning in older adults, such as through stimulating activities or emotional support, which in turn may foster positive well-being [63, 64]. This suggests that family dynamics play a crucial role in shaping the relationship between cognitive abilities and emotional well-being in older adults. The centrality of “Depressed mood” across all three waves of the contemporaneous networks may be closely linked to societal expectations of aging. Older adults often face significant pressure to maintain independence, health, and productivity, which can lead to heightened emotional distress when these expectations are not met [65]. This societal pressure may exacerbate feelings of inadequacy, loneliness, or failure, thereby reinforcing the prominence of “Depressed mood” in their emotional and psychological networks. Furthermore, the stigma associated with aging and mental health issues can discourage older adults from seeking support, perpetuating a cycle of depression and cognitive decline [66]. However, this study has not thoroughly explored the potential influence of Chinese cultural values on the associations between depression symptoms and cognitive functions. Future research should further focus on this aspect to comprehensively reveal how cultural factors may amplify or mitigate these relationships.

Although this study provides some insights into the longitudinal developmental processes between cognitive function and depression symptoms in older adults, there are several limitations to consider. First, the findings of network analysis should be interpreted as generating hypotheses rather than verifying causal relationships [29], so the results of this study should be interpreted with caution. Future research will integrate quantitative network analysis with qualitative data to comprehensively evaluate the validity of hypotheses and the strength of causal relationships. Second, due to the inconsistency in cognitive function issues across different survey waves, although efforts have been made to extract five commonly measured cognitive abilities, this variation may affect the comparability and consistency of the assessment results. Future research will employ the same cognitive function assessment questionnaire across different time points to ensure the comparability of research findings. Third, this study excluded elderly participants with severe cognitive impairments who were unable to self-report information, which may introduce selection bias and affect the generalizability of the findings. Future studies could incorporate proxy reports or caregiver-assisted assessments for participants with cognitive impairments, thereby reducing the exclusion of vulnerable populations. Fourth, the data collection method in this study relied on self-reported questionnaires, which may introduce potential information bias during the survey process. For example, self-reported measures of cognitive function may underestimate true cognitive abilities, as they can be influenced by participants' subjective perceptions, lack of self-awareness, or social desirability. While self-reported measures are widely used in large-scale studies, they may not fully capture the complexity of cognitive functioning. Future research should incorporate more objective measures, such as clinical diagnoses or neuroimaging, to validate the findings and provide a more comprehensive understanding of the depression–cognition relationship. Fifth, this study did not adopt a more rigorous control strategy for covariates such as age, sex, marital status, and residence. Existing research has employed methods where these covariates are regressed onto core variables, and the residuals are used in CLPN analysis to better control for the influence of covariates [67]. In our future research, we will employ this method to mitigate the influence of covariates on the relationships between specific symptoms. Sixth, this study did not examine the potential moderating role of gender in the dynamic relationship between depression symptoms and cognitive functions among Chinese older adults. Given established evidence indicating gender differences [62, 68], future studies should employ multigroup CLPN analysis to uncover unique psychopathological network features across genders among Chinese older adults. Finally, this study did not account for potential confounding factors such as socioeconomic status, physical health, chronic disease burden, and social support networks, which may introduce limitations in interpreting the observed relationships between depression symptoms and cognitive function. Future research should explore the role of these factors in influencing the dynamic interplay between depression and cognition.

## 5. Conclusion

This study leveraged data from the CHARLS to explore the dynamic interactions between depression symptoms and cognitive functions in Chinese older adults. The longitudinal and bidirectional associations between specific depression symptoms and distinct cognitive functions have enriched the evidence for the scar theory model and the cognitive vulnerability model. By uncovering the most susceptible and influential symptoms in dynamic temporal networks, this study also provides new directions for the early identification of older adults at high risk of depression symptoms and cognitive decline. Overall, this research advances our understanding of the complex interplay between depression and cognitive decline in aging populations and provides actionable insights for evidence-based interventions and policies. Future studies should integrate quantitative and qualitative data to strengthen causal validation and assess the generalizability of findings across diverse cultural contexts.

## Figures and Tables

**Table 1:** Demographic information (*N* = 5203).

Variable	*N* (%)
Gender	
Male	2545 (48.91)
Female	2658 (51.09)
Age in 2020	
65–69 years	2413 (46.38)
70–74 years	1493 (28.69)
75–79 years	844 (16.22)
≥80 years	453 (8.71)
Marital status	
Married	3669 (70.52)
Divorced	39 (0.75)
Widowed	1295 (24.89)
Never married	28 (0.54)
Other	172 (3.31)
Residence	
Urban	1031 (19.82)
Rural	3674 (70.61)
Urban–rural fringe areas	493 (9.48)
Missing data	5 (0.09)

**Table 2:** Mean (*M*), standard deviation (SD), skewness, and kurtosis of cognitive function and depression symptoms (*N* = 5203).

Wave	Items	*M*	SD	Skewness	Kurtosis
Wave 1	Orientation	3.47	1.57	−0.81	−0.50
	Memory	3.36	1.86	−0.06	−0.17
	Attention	2.62	1.99	0.09	−1.65
	Drawing	0.54	0.50	−0.17	−1.97
	Recall	2.32	1.97	0.40	−0.53
	Bothered	1.86	1.12	0.89	−0.75
	Mind distraction	1.87	1.13	0.87	−0.81
	Depressed mood	1.88	1.11	0.84	−0.81
	Exhaust	1.93	1.19	0.78	−1.04
	Hope	2.45	1.31	0.05	−1.73
	Fear	1.32	0.79	2.49	4.92
	Sleeplessness	2.05	1.23	0.61	−1.30
	Happy	2.89	1.23	−0.56	−1.34
	Loneliness	1.58	1.03	1.50	0.68
	Cannot continue	1.38	0.85	2.18	3.38

Wave 2	Orientation	3.34	1.47	−0.59	−0.62
	Memory	2.17	1.97	0.56	−0.40
	Attention	2.03	2.00	0.49	−1.40
	Drawing	0.41	0.49	0.37	−1.87
	Recall	2.70	2.74	0.54	−0.97
	Bothered	1.86	1.11	0.89	−0.71
	Mind distraction	1.92	1.11	0.78	−0.88
	Depressed mood	1.94	1.11	0.74	−0.93
	Exhaust	2.07	1.21	0.58	−1.29
	Hope	2.50	1.30	−0.01	−1.71
	Fear	1.40	0.87	2.07	2.95
	Sleeplessness	2.14	1.24	0.48	−1.43
	Happy	2.94	1.20	−0.62	−1.23
	Loneliness	1.67	1.09	1.28	0.04
	Cannot continue	1.49	0.95	1.74	1.59

Wave 3	Orientation	3.31	1.47	−0.60	−0.59
	Memory	2.76	1.85	0.03	−0.64
	Attention	2.22	2.01	0.32	−1.52
	Drawing	0.21	0.41	1.39	−0.06
	Recall	3.76	2.70	−0.10	−1.10
	Bothered	1.96	1.13	0.68	−1.07
	Mind distraction	2.05	1.16	0.54	−1.26
	Depressed mood	1.98	1.13	0.64	−1.12
	Exhaust	2.14	1.22	0.44	−1.44
	Hope	2.37	1.27	0.14	−1.66
	Fear	1.44	0.90	1.88	2.10
	Sleeplessness	2.13	1.23	0.46	−1.44
	Happy	2.83	1.22	−0.48	−1.37
	Loneliness	1.70	1.09	1.19	−0.20
	Cannot continue	1.54	0.99	1.56	0.91

## Data Availability

The evaluation scales and data used in this study for depression symptoms and cognitive function among Chinese elderly were obtained from the China Health and Retirement Longitudinal Study (CHARLS). CHARLS is an open database, meaning that we can obtain the data through an application and use it for relevant scientific research. The dataset supporting the conclusions of this article is available at http://charls.pku.edu.cn/.

## Supporting Information

Supporting Information Table S1. Partial correlation coefficient between undirected edges of nodes in the network (wave 1, *N* = 5203). Table S2. Partial correlation coefficient between undirected edges of nodes in the network (wave 2, *N* = 5203). Table S3. Partial correlation coefficient between undirected edges of nodes in the network (wave 3, *N* = 5203). Table S4. Centrality metrics (strength and expected influence) values of the contemporaneous networks (*N* = 5203). Table S5. Partial directed correlations (standardized beta coefficients) of the temporal network (wave 1→wave 2, *N* = 5203). Table S6. Partial directed correlations (standardized beta coefficients) of the temporal network (wave 2→wave 3, *N* = 5203). Table S7. Centrality metrics (in-prediction [predictability] and out-prediction [influence]) of the temporal network (*N* = 5203). Figure S1. Flowchart of participant selection process. Figure S2. Three waves (2015, 2018, 2020) of cognitive function and depression symptoms network analyses of nodal strength and expected impact. Figure S3. Three waves (2015, 2018, 2020) of cognitive function and depression symptoms network analyses of nodal bridge strength and bridge expected impact. Figure S4. Bootstrapped confidence intervals of the edge weights in the network of cognitive function and depression symptoms at three different waves. Figure S5. Centrality stability in the network using case-drop bootstrapping at three different waves. Figure S6. Edge weight difference tests for the network of cognitive function and depression symptoms at three different waves. Figure S7. Node centrality difference tests for the network of cognitive function and depression symptoms at three different waves. Figure S8. The autoregressive impact of network nodes from wave 1 to wave 2 and from wave 2 to wave 3. Figure S9. Accuracy of the edge-weight estimates of the temporal network. Figure S10. Stability of central indices of the temporal network.
