# Supplementary material for: Longitudinal Associations Between Depression Symptoms and Cognitive Functions in Chinese Older Adults: A Cross-Lagged Panel Network Analysis
Source: Depress Anxiety. 2025 Oct 10;2025:3984020. doi: 10.1155/da/3984020 (PMC12534153; doi:10.1155/da/3984020)
Supplement: Supporting Information — Table S1. Partial correlation coefficient between undirected edges of nodes in the network (wave 1, N = 5203). Table S2. Partial correlation coefficient between undirected edges of nodes in the network (wave 2, N = 5203). Table S3. Partial correlation coefficient between undirected edges of nodes in the network (wave 3, N = 5203). Table S4. Centrality metrics (strength and expected influence) values of the contemporaneous networks (N = 5203). Table S5. Partial directed correlations (standardized beta coefficients) of the temporal network (wave 1→wave 2, N = 5203). Table S6. Partial directed correlations (standardized beta coefficients) of the temporal network (wave 2→wave 3, N = 5203). Table S7. Centrality metrics (in-prediction [predictability] and out-prediction [influence]) of the temporal network (N = 5203). Figure S1. Flowchart of participant selection process. Figure S2. Three waves (2015, 2018, 2020) of cognitive function and depression symptoms network analyses of nodal strength and expected impact. Figure S3. Three waves (2015, 2018, 2020) of cognitive function and depression symptoms network analyses of nodal bridge strength and bridge expected impact. Figure S4. Bootstrapped confidence intervals of the edge weights in the network of cognitive function and depression symptoms at three different waves. Figure S5. Centrality stability in the network using case-drop bootstrapping at three different waves. Figure S6. Edge weight difference tests for the network of cognitive function and depression symptoms at three different waves. Figure S7. Node centrality difference tests for the network of cognitive function and depression symptoms at three different waves. Figure S8. The autoregressive impact of network nodes from wave 1 to wave 2 and from wave 2 to wave 3. Figure S9. Accuracy of the edge-weight estimates of the temporal network. Figure S10. Stability of central indices of the temporal network. [file 3984020.f1.docx]

Supplementary Materials

Table S1. Partial correlation coefficient between undirected edges of nodes in the network (Wave 1, N = 5,203)

Table S2. Partial correlation coefficient between undirected edges of nodes in the network (Wave 2, N = 5,203)

Table S3. Partial correlation coefficient between undirected edges of nodes in the network (Wave 3, N = 5,203)

Table S4. Centrality metrics (strength and expected influence) values of the contemporaneous networks (N = 5,203)

Table S5. Partial directed correlations (standardized beta coefficients) of temporal network (Wave1→Wave2, N = 5,203)

Table S6. Partial directed correlations (standardized beta coefficients) of temporal network (Wave2→Wave3, N = 5,203)

Table S7. Centrality metrics (in-prediction (predictability) and out-prediction (influence)) of temporal network (N = 5,203)

Fig. S1. Flowchart of participant selection process

Fig. S2. Three waves (2015, 2018, 2020) of cognitive function and depression symptoms network analyses of nodal strength and expected impact

Fig. S3. Three waves (2015, 2018, 2020) of cognitive function and depression symptoms network analyses of nodal bridge strength and bridge expected impact

Fig. S4. Bootstrapped confidence intervals of the edge weights in the network of cognitive function and depression symptoms at three different waves

Fig. S5. Centrality stability in the network using case-drop bootstrapping at three different waves

Fig. S6. Edge weight difference tests for the network of cognitive function and depression symptoms at three different waves

Fig. S7. Node centrality difference tests for the network of cognitive function and depression symptoms at three different waves

Fig. S8. The autoregressive impact of network nodes from Wave1 to Wave2 and from Wave2 to Wave3

Fig. S9. Accuracy of the edge-weight estimates of the temporal network

Fig. S10. Stability of central indices of the temporal network

|  | **Orient** | **Memo** | **Atten** | **Draw** | **Recal** | **Bother** | **Min_dis** | **Dep** | **Exha** | **Hop** | **Fea** | **Sleepl** | **Hap** | **Lone** | **Not_Con** |
| --- | --- | --- | --- | --- | --- | --- | --- | --- | --- | --- | --- | --- | --- | --- | --- |
| **Orien** | 0.000 |  |  |  |  |  |  |  |  |  |  |  |  |  |  |
| **Memo** | 0.115 | 0.000 |  |  |  |  |  |  |  |  |  |  |  |  |  |
| **Atten** | 0.281 | 0.095 | 0.000 |  |  |  |  |  |  |  |  |  |  |  |  |
| **Draw** | 0.209 | 0.082 | 0.209 | 0.000 |  |  |  |  |  |  |  |  |  |  |  |
| **Recal** | 0.067 | 0.645 | 0.036 | 0.0480 | 0.000 |  |  |  |  |  |  |  |  |  |  |
| **Bother** | 0.000 | 0.000 | 0.005 | 0.000 | 0.000 | 0.000 |  |  |  |  |  |  |  |  |  |
| **Min_dis** | 0.000 | -0.010 | -0.010 | -0.004 | 0.000 | 0.153 | 0.000 |  |  |  |  |  |  |  |  |
| **Dep** | -0.026 | 0.000 | 0.000 | -0.001 | 0.000 | 0.277 | 0.195 | 0.000 |  |  |  |  |  |  |  |
| **Exha** | -0.04611 | -0.010 | 0.000 | -0.017 | 0.000 | 0.122 | 0.174 | 0.234 | 0.000 |  |  |  |  |  |  |
| **Hop** | 0.04617 | 0.0318 | 0.000 | 0.038 | 0.016 | 0.006 | 0.031 | 0.000 | 0.000 | 0.000 |  |  |  |  |  |
| **Fea** | 0.000 | 0.000 | -0.011 | 0.000 | 0.000 | 0.050 | 0.072 | 0.065 | 0.061 | 0.009 | 0.000 |  |  |  |  |
| **Sleepl** | -0.033 | 0.000 | -0.012 | -0.009 | 0.000 | 0.098 | 0.030 | 0.065 | 0.065 | 0.010 | 0.088 | 0.000 |  |  |  |
| **Hap** | 0.029 | 0.000 | 0.000 | 0.002 | 0.008 | -0.088 | -0.006 | -0.108 | -0.031 | 0.321 | -0.011 | -0.038 | 0.000 |  |  |
| **Lone** | -0.018 | -0.008 | -0.017 | -0.015 | -0.006 | 0.030 | 0.041 | 0.129 | 0.062 | 0.000 | 0.134 | 0.066 | -0.099 | 0.000 |  |
| **Not_Con** | -0.031 | -0.023 | -0.036 | 0.000 | -0.004 | 0.048 | 0.049 | 0.048 | 0.101 | 0.000 | 0.169 | 0.063 | -0.058 | 0.253 | 0.000 |

Table S1. Partial correlation coefficient between undirected edges of nodes in the network (Wave 1, N = 5,203)

Table S2. Partial correlation coefficient between undirected edges of nodes in the network (Wave 2, N = 5,203)

|  | **Orient** | **Memo** | **Atten** | **Draw** | **Recal** | **Bother** | **Min_dis** | **Dep** | **Exha** | **Hop** | **Fea** | **Sleepl** | **Hap** | **Lone** | **Not_Con** |
| --- | --- | --- | --- | --- | --- | --- | --- | --- | --- | --- | --- | --- | --- | --- | --- |
| **Orien** | 0.000 |  |  |  |  |  |  |  |  |  |  |  |  |  |  |
| **Memo** | 0.116 | 0.000 |  |  |  |  |  |  |  |  |  |  |  |  |  |
| **Atten** | 0.259 | 0.117 | 0.000 |  |  |  |  |  |  |  |  |  |  |  |  |
| **Draw** | 0.131 | 0.072 | 0.183 | 0.000 |  |  |  |  |  |  |  |  |  |  |  |
| **Recal** | 0.165 | 0.546 | 0.162 | 0.113 | 0.000 |  |  |  |  |  |  |  |  |  |  |
| **Bother** | 0.000 | 0.000 | 0.000 | -0.006 | 0.000 | 0.000 |  |  |  |  |  |  |  |  |  |
| **Min_dis** | -0.009 | 0.000 | 0.000 | 0.000 | 0.000 | 0.177 | 0.000 |  |  |  |  |  |  |  |  |
| **Dep** | 0.000 | 0.000 | 0.000 | 0.000 | 0.000 | 0.294 | 0.232 | 0.000 |  |  |  |  |  |  |  |
| **Exha** | -0.0361 | -0.013 | 0.000 | 0.000 | -0.028 | 0.085 | 0.163 | 0.269 | 0.000 |  |  |  |  |  |  |
| **Hop** | 0.006 | 0.034 | 0.026 | 0.000 | 0.0362 | 0.027 | 0.019 | 0.026 | 0.007 | 0.000 |  |  |  |  |  |
| **Fea** | -0.017 | 0.000 | -0.008 | 0.000 | 0.000 | 0.071 | 0.022 | 0.0701 | 0.061 | 0.024 | 0.000 |  |  |  |  |
| **Sleepl** | 0.000 | -0.013 | -0.022 | -0.008 | 0.000 | 0.059 | 0.051 | 0.064 | 0.088 | 0.000 | 0.087 | 0.000 |  |  |  |
| **Hap** | 0.027 | 0.000 | 0.000 | 0.0152 | 0.000 | -0.087 | -0.002 | -0.101 | -0.023 | 0.307 | 0.000 | -0.079 | 0.000 |  |  |
| **Lone** | -0.031 | -0.012 | -0.014 | 0.000 | -0.005 | 0.035 | 0.069 | 0.086 | 0.051 | 0.000 | 0.123 | 0.075 | -0.114 | 0.000 |  |
| **Not_Con** | -0.004 | -0.011 | -0.014 | -0.006 | -0.029 | 0.053 | 0.034 | 0.047 | 0.106 | 0.000 | 0.224 | 0.049 | -0.057 | 0.280 | 0.000 |

Table S3. Partial correlation coefficient between undirected edges of nodes in the network (Wave 3, N = 5,203)

|  | **Orient** | **Memo** | **Atten** | **Draw** | **Recal** | **Bother** | **Min_dis** | **Dep** | **Exha** | **Hop** | **Fea** | **Sleepl** | **Hap** | **Lone** | **Not_Con** |
| --- | --- | --- | --- | --- | --- | --- | --- | --- | --- | --- | --- | --- | --- | --- | --- |
| **Orien** | 0.000 |  |  |  |  |  |  |  |  |  |  |  |  |  |  |
| **Memo** | 0.130 | 0.000 |  |  |  |  |  |  |  |  |  |  |  |  |  |
| **Atten** | 0.308 | 0.154 | 0.000 |  |  |  |  |  |  |  |  |  |  |  |  |
| **Draw** | 0.100 | 0.045 | 0.118 | 0.000 |  |  |  |  |  |  |  |  |  |  |  |
| **Recal** | 0.135 | 0.532 | 0.126 | 0.048 | 0.000 |  |  |  |  |  |  |  |  |  |  |
| **Bother** | 0.000 | 0.000 | 0.000 | 0.000 | 0.022 | 0.000 |  |  |  |  |  |  |  |  |  |
| **Min_dis** | 0.000 | 0.000 | 0.000 | -0.002 | -0.013 | 0.201 | 0.000 |  |  |  |  |  |  |  |  |
| **Dep** | 0.000 | 0.000 | 0.000 | 0.000 | 0.000 | 0.262 | 0.245 | 0.000 |  |  |  |  |  |  |  |
| **Exha** | -0.040 | -0.002 | 0.000 | 0.000 | -0.017 | 0.084 | 0.175 | 0.272 | 0.000 |  |  |  |  |  |  |
| **Hop** | 0.0533 | 0.013 | 0.015 | 0.049 | 0.042 | 0.009 | 0.056 | 0.048 | 0.019 | 0.000 |  |  |  |  |  |
| **Fea** | -0.018 | -0.006 | -0.025 | -0.003 | 0.000 | 0.064 | 0.055 | 0.048 | 0.059 | 0.055 | 0.000 |  |  |  |  |
| **Sleepl** | 0.000 | 0.000 | -0.021 | 0.000 | -0.005 | 0.098 | 0.055 | 0.072 | 0.087 | 0.000 | 0.118 | 0.000 |  |  |  |
| **Hap** | 0.0531 | 0.016 | 0.013 | 0.009 | 0.027 | -0.095 | 0.000 | -0.054 | -0.018 | 0.307 | -0.036 | -0.024 | 0.000 |  |  |
| **Lone** | -0.020 | -0.001 | -0.009 | -0.002 | -0.034 | 0.042 | 0.000 | 0.082 | 0.077 | 0.002 | 0.148 | 0.083 | -0.062 | 0.000 |  |
| **Not_Con** | -0.009 | -0.012 | -0.027 | -0.020 | -0.022 | 0.040 | 0.052 | 0.094 | 0.053 | 0.016 | 0.165 | 0.072 | -0.048 | 0.324 | 0.000 |

Table S4. Centrality metrics (strength and expected influence) values of the contemporaneous networks (N = 5,203)

| Variables | Wave 1 | | Wave 2 | | Wave 3 | |
| --- | --- | --- | --- | --- | --- | --- |
|  | Strength | Expected Influence | Strength | Expected Influence | Strength | Expected Influence |
| Orientation | 0.546 | -0.025 | -0.133 | -0.076 | 0.151 | 0.112 |
| Memory | 1.241 | 1.350 | 0.575 | 0.791 | 0.396 | 0.884 |
| Attention | -0.573 | -0.250 | -0.119 | 0.235 | -0.128 | -0.067 |
| Drawing | -1.037 | -0.245 | -1.567 | -0.496 | -2.474 | -1.424 |
| Recall | 0.127 | 0.891 | 1.376 | 1.255 | 1.027 | 0.761 |
| Bothered | 0.395 | 0.422 | 0.360 | 0.307 | 0.434 | 0.261 |
| Mind distraction | -0.205 | 0.490 | -0.246 | 0.489 | 0.088 | 0.691 |
| Depressed mood | 2.009 | 1.179 | 1.934 | 1.356 | 1.887 | 1.760 |
| Exhaust | 0.669 | 0.494 | 0.568 | 0.393 | 0.362 | 0.367 |
| Hope | -1.778 | -0.391 | -1.661 | -0.420 | -0.862 | 0.075 |
| Fear | -0.824 | 0.109 | -0.629 | 0.123 | -0.221 | -0.189 |
| Sleeplessness | -1.361 | -0.873 | -1.231 | -1.031 | -1.135 | -0.582 |
| Happy | -0.059 | -2.869 | -0.068 | -2.789 | -0.426 | -2.531 |
| Loneliness | 0.414 | -0.201 | 0.368 | -0.314 | 0.259 | -0.163 |
| Cannot continue | 0.436 | -0.082 | 0.473 | 0.177 | 0.644 | 0.046 |

Table S5. Partial directed correlations (standardized beta coefficients) of temporal network (Wave1→Wave2, N = 5,203)

|  | **Orient** | **Memo** | **Atten** | **Draw** | **Recal** | **Bother** | **Min_dis** | **Dep** | **Exha** | **Hop** | **Fea** | **Sleepl** | **Hap** | **Lone** | **Not_Con** |
| --- | --- | --- | --- | --- | --- | --- | --- | --- | --- | --- | --- | --- | --- | --- | --- |
| **Orien** | 0.380 | 0.196 | 0.178 | 0.043 | 0.323 | 0.000 | -0.006 | -0.010 | -0.034 | 0.030 | -0.007 | 0.000 | 0.043 | -0.004 | 0.000 |
| **Memo** | 0.062 | 0.185 | 0.068 | 0.013 | 0.225 | -0.013 | -0.023 | -0.005 | -0.029 | 0.002 | -0.005 | -0.008 | 0.014 | -0.020 | -0.032 |
| **Atten** | 0.102 | 0.113 | 0.285 | 0.032 | 0.172 | -0.001 | -0.008 | -0.007 | -0.008 | 0.000 | -0.005 | -0.004 | 0.000 | -0.014 | -0.006 |
| **Draw** | 0.372 | 0.541 | 0.488 | 0.232 | 0.784 | -0.023 | 0.037 | -0.040 | 0.000 | 0.083 | -0.007 | 0.000 | -0.052 | -0.034 | -0.043 |
| **Recal** | 0.027 | 0.132 | 0.072 | 0.013 | 0.237 | 0.000 | 0.000 | 0.000 | 0.000 | 0.027 | 0.000 | 0.000 | 0.000 | -0.009 | 0.000 |
| **Bother** | 0.000 | -0.006 | 0.000 | -0.008 | 0.000 | 0.133 | 0.069 | 0.097 | 0.062 | -0.016 | 0.049 | 0.048 | -0.048 | 0.028 | 0.037 |
| **Min_dis** | 0.000 | 0.000 | 0.000 | 0.004 | 0.000 | 0.025 | 0.053 | 0.017 | 0.022 | -0.008 | 0.019 | 0.002 | -0.019 | 0.000 | 0.007 |
| **Dep** | -0.001 | -0.004 | -0.019 | 0.005 | 0.000 | 0.093 | 0.059 | 0.117 | 0.082 | -0.011 | 0.019 | 0.025 | -0.047 | 0.042 | 0.070 |
| **Exha** | 0.000 | -0.020 | 0.000 | 0.000 | -0.024 | 0.051 | 0.095 | 0.067 | 0.169 | -0.002 | 0.033 | 0.041 | -0.038 | 0.043 | 0.044 |
| **Hop** | 0.000 | 0.006 | 0.000 | 0.000 | 0.000 | 0.000 | 0.000 | -0.012 | -0.027 | 0.164 | 0.000 | -0.035 | 0.050 | -0.014 | -0.016 |
| **Fea** | -0.006 | 0.000 | -0.002 | -0.010 | -0.056 | 0.028 | 0.020 | 0.053 | 0.025 | 0.000 | 0.198 | 0.020 | -0.002 | 0.040 | 0.072 |
| **Sleepl** | 0.000 | 0.000 | 0.009 | 0.009 | 0.000 | 0.040 | 0.045 | 0.033 | 0.052 | 0.000 | 0.019 | 0.326 | -0.050 | 0.034 | 0.017 |
| **Hap** | 0.009 | 0.000 | 0.000 | 0.000 | 0.000 | -0.047 | -0.038 | -0.044 | -0.045 | 0.041 | -0.028 | -0.044 | 0.174 | -0.044 | -0.043 |
| **Lone** | -0.004 | -0.020 | 0.000 | 0.000 | -0.051 | 0.051 | 0.030 | 0.069 | 0.037 | 0.000 | 0.044 | 0.000 | -0.074 | 0.225 | 0.064 |
| **Not_Con** | -0.047 | -0.040 | -0.017 | -0.010 | -0.015 | 0.062 | 0.003 | 0.053 | 0.060 | -0.029 | 0.006 | 0.019 | -0.055 | 0.077 | 0.123 |

Table S6. Partial directed correlations (standardized beta coefficients) of temporal network (Wave2→Wave3, N = 5,203)

|  | **Orient** | **Memo** | **Atten** | **Draw** | **Recal** | **Bother** | **Min_dis** | **Dep** | **Exha** | **Hop** | **Fea** | **Sleepl** | **Hap** | **Lone** | **Not_Con** |
| --- | --- | --- | --- | --- | --- | --- | --- | --- | --- | --- | --- | --- | --- | --- | --- |
| **Orien** | 0.089 | -0.043 | -0.023 | -0.029 | -0.010 | 0.000 | 0.083 | 0.031 | 0.082 | 0.082 | -0.022 | 0.036 | 0.044 | -0.105 | 0.053 |
| **Memo** | 0.000 | 0.460 | 0.176 | 0.246 | 0.030 | 0.254 | 0.018 | 0.000 | 0.000 | 0.000 | 0.040 | -0.024 | 0.002 | 0.043 | -0.008 |
| **Atten** | 0.000 | 0.042 | 0.155 | 0.038 | 0.000 | 0.154 | 0.000 | 0.000 | 0.000 | 0.000 | 0.000 | 0.000 | 0.007 | 0.000 | 0.000 |
| **Draw** | -0.002 | 0.079 | 0.078 | 0.303 | 0.018 | 0.087 | 0.006 | 0.000 | 0.000 | 0.000 | 0.006 | 0.000 | 0.000 | 0.000 | 0.000 |
| **Recal** | -0.007 | 0.206 | 0.090 | 0.409 | 0.138 | 0.161 | 0.023 | 0.000 | 0.000 | 0.000 | 0.084 | -0.032 | 0.000 | 0.047 | 0.000 |
| **Bother** | -0.011 | 0.056 | 0.164 | 0.087 | 0.009 | 0.316 | -0.005 | -0.008 | 0.000 | -0.008 | 0.033 | 0.000 | 0.000 | 0.013 | -0.008 |
| **Min_dis** | 0.033 | 0.000 | 0.005 | -0.013 | -0.004 | 0.000 | 0.094 | 0.021 | 0.033 | 0.000 | -0.003 | 0.006 | 0.010 | -0.014 | 0.011 |
| **Dep** | 0.029 | 0.000 | 0.021 | 0.000 | 0.000 | 0.000 | 0.010 | 0.084 | 0.044 | 0.058 | 0.000 | 0.019 | 0.011 | 0.000 | 0.000 |
| **Exha** | 0.057 | -0.001 | 0.000 | 0.000 | 0.000 | 0.000 | 0.068 | 0.062 | 0.094 | 0.040 | -0.010 | 0.015 | 0.006 | -0.005 | 0.021 |
| **Hop** | 0.072 | -0.016 | -0.023 | 0.000 | 0.000 | 0.000 | 0.046 | 0.054 | 0.068 | 0.186 | 0.000 | 0.008 | 0.034 | -0.066 | 0.010 |
| **Fea** | 0.000 | 0.009 | 0.014 | 0.000 | 0.000 | 0.025 | 0.009 | -0.003 | 0.000 | -0.018 | 0.175 | 0.000 | 0.000 | 0.050 | -0.010 |
| **Sleepl** | 0.236 | -0.029 | -0.014 | 0.000 | 0.000 | 0.000 | 0.042 | 0.044 | 0.005 | 0.000 | 0.013 | 0.159 | 0.005 | -0.003 | 0.039 |
| **Hap** | 0.023 | 0.005 | 0.044 | 0.000 | 0.000 | 0.000 | 0.063 | 0.013 | 0.035 | 0.040 | -0.009 | 0.023 | 0.319 | -0.006 | 0.011 |
| **Lone** | -0.017 | 0.000 | 0.011 | 0.009 | 0.000 | 0.000 | -0.060 | -0.019 | -0.045 | -0.021 | 0.043 | 0.000 | -0.033 | 0.169 | -0.024 |
| **Not_Con** | 0.221 | 0.000 | 0.000 | 0.000 | 0.000 | 0.000 | 0.010 | 0.001 | 0.005 | 0.002 | -0.012 | 0.012 | 0.000 | -0.047 | 0.174 |

Table S7. Centrality metrics (in-prediction (predictability) and out-prediction (influence)) of temporal network (N = 5,203)

| Variables | Wave 1→Wave 2 | | Wave 2→ Wave 3 | |
| --- | --- | --- | --- | --- |
|  | In-prediction | Out-prediction | In-prediction | Out-prediction |
| Orientation | 0.514 | 0.752 | 0.632 | 0.180 |
| Memory | 0.898 | 0.250 | 0.308 | 0.771 |
| Attention | 0.777 | 0.366 | 0.542 | 0.240 |
| Drawing | 0.093 | 2.106 | 0.747 | 0.272 |
| Recall | 1.358 | 0.262 | 0.044 | 0.980 |
| Bothered | 0.264 | 0.313 | 0.681 | 0.324 |
| Mind distraction | 0.283 | 0.069 | 0.306 | 0.085 |
| Depressed mood | 0.271 | 0.313 | 0.196 | 0.191 |
| Exhaust | 0.198 | 0.291 | 0.226 | 0.255 |
| Hope | 0.119 | -0.047 | 0.175 | 0.186 |
| Fear | 0.136 | 0.183 | 0.164 | 0.075 |
| Sleeplessness | 0.065 | 0.209 | 0.064 | 0.337 |
| Happy | -0.276 | -0.283 | 0.085 | 0.241 |
| Loneliness | 0.124 | 0.146 | -0.093 | -0.157 |
| Cannot continue | 0.172 | 0.068 | 0.095 | 0.192 |

Fig. S1. Flowchart of participant selection process


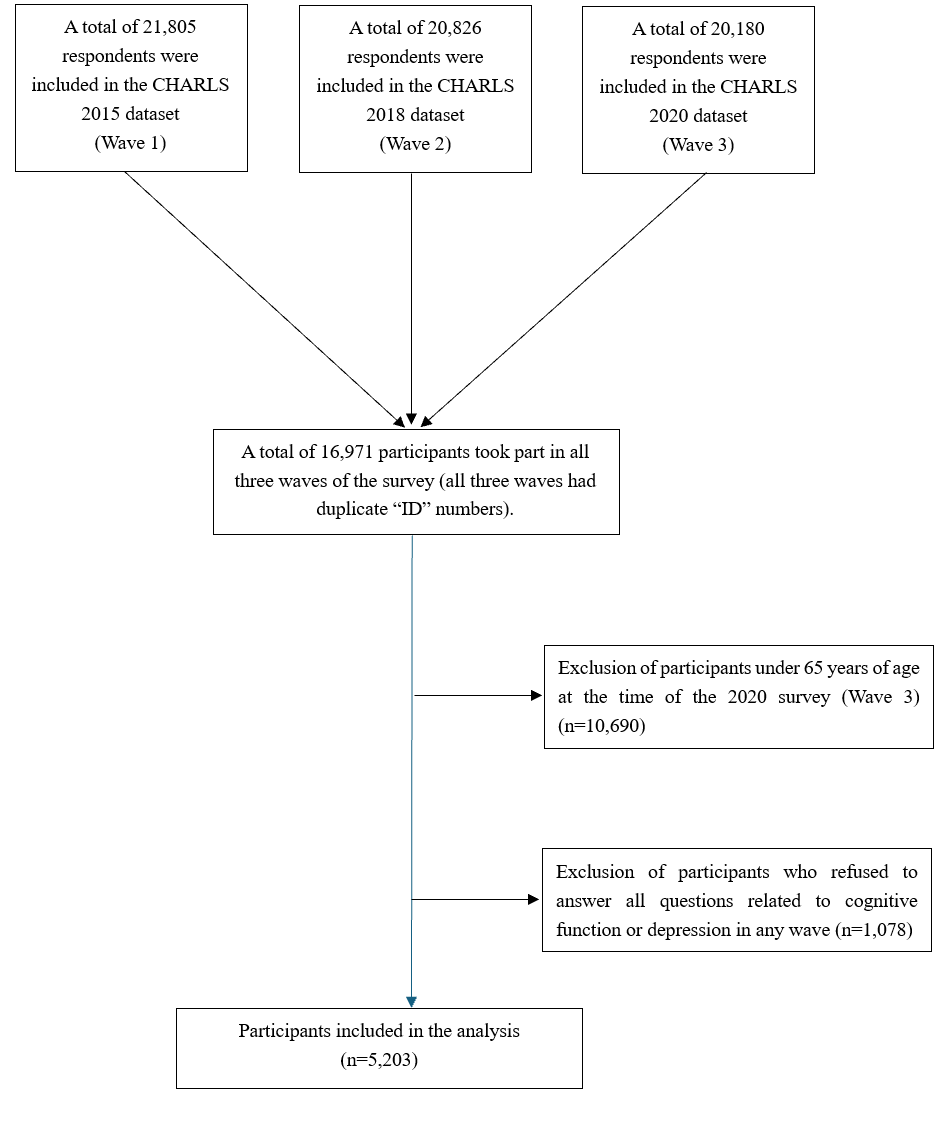


Fig. S2. Three waves (2015, 2018, 2020) of cognitive function and depression symptoms network analyses of nodal strength and expected impact


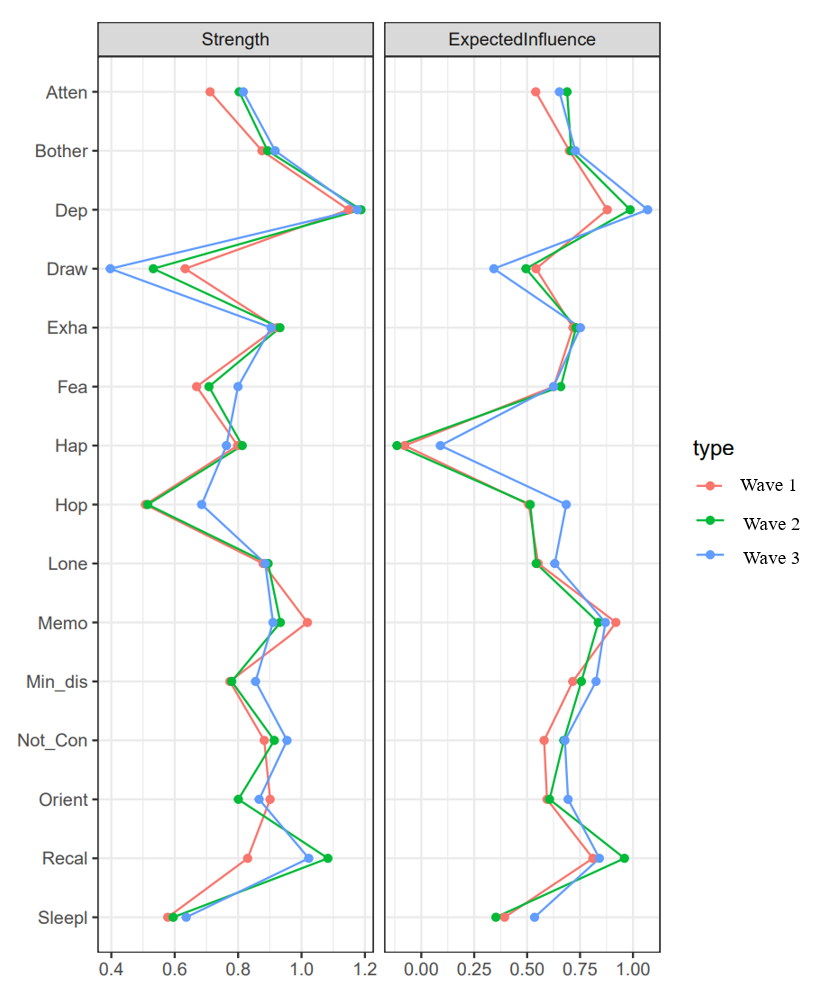


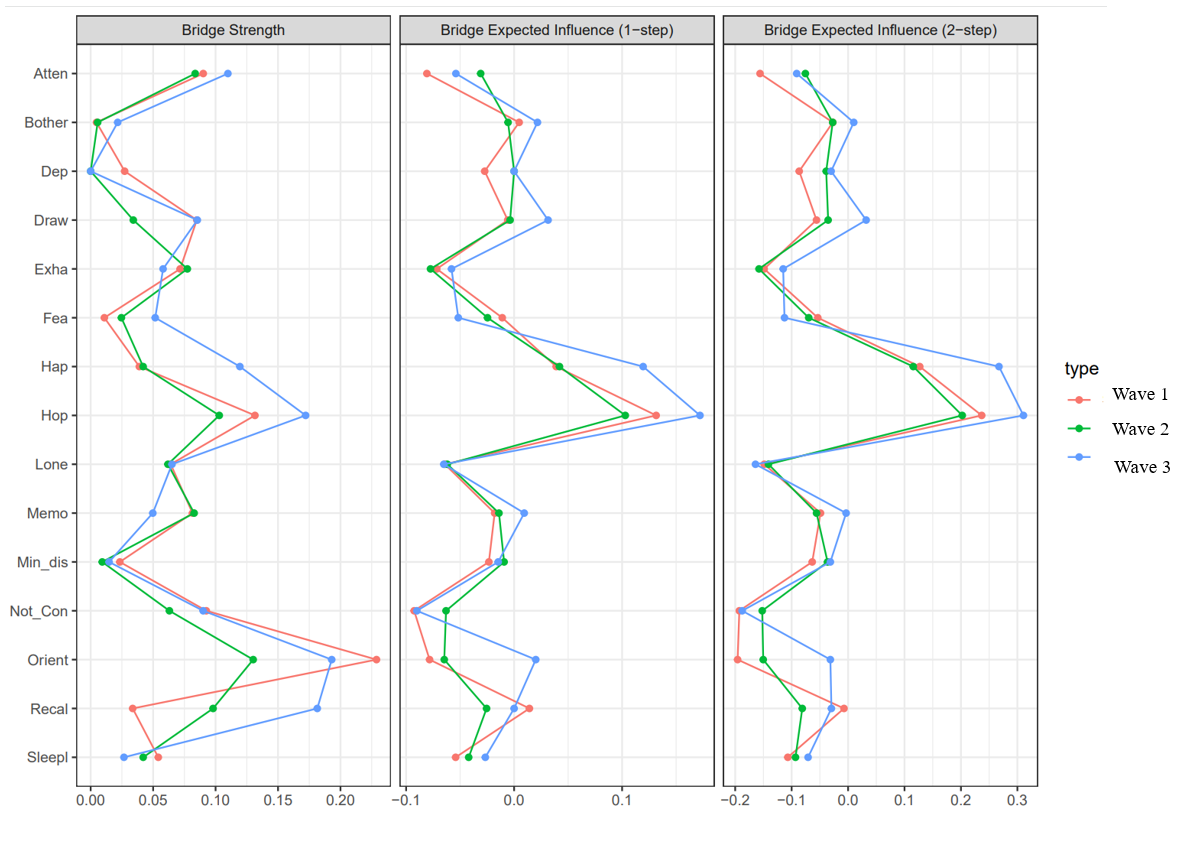
Fig. S3. Three waves (2015, 2018, 2020) of cognitive function and depression symptoms network analyses of nodal bridge strength and bridge expected impact

Fig. S4. Bootstrapped confidence intervals of the edge weights in the network of cognitive function and depression symptoms at three different waves


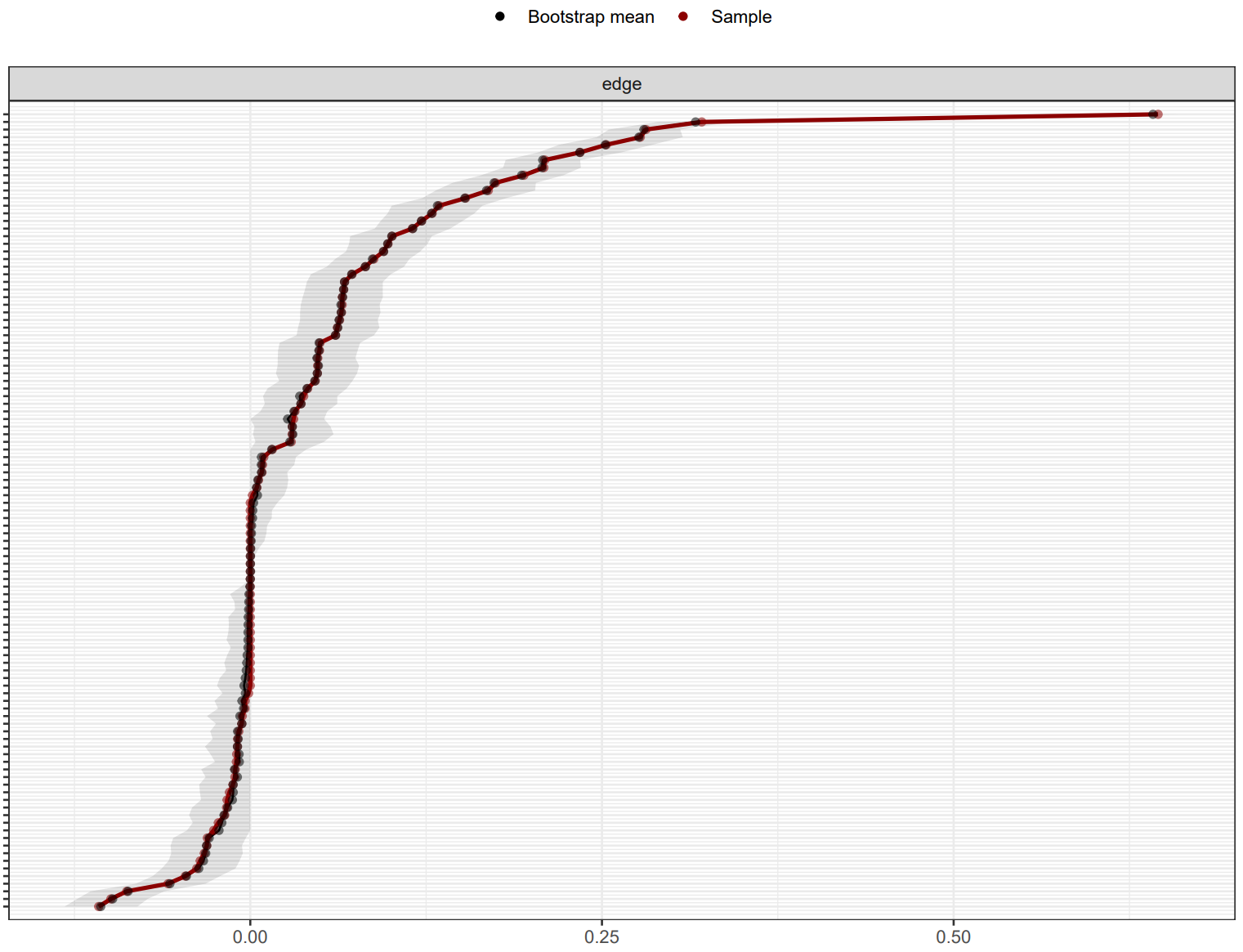


**Wave 1**


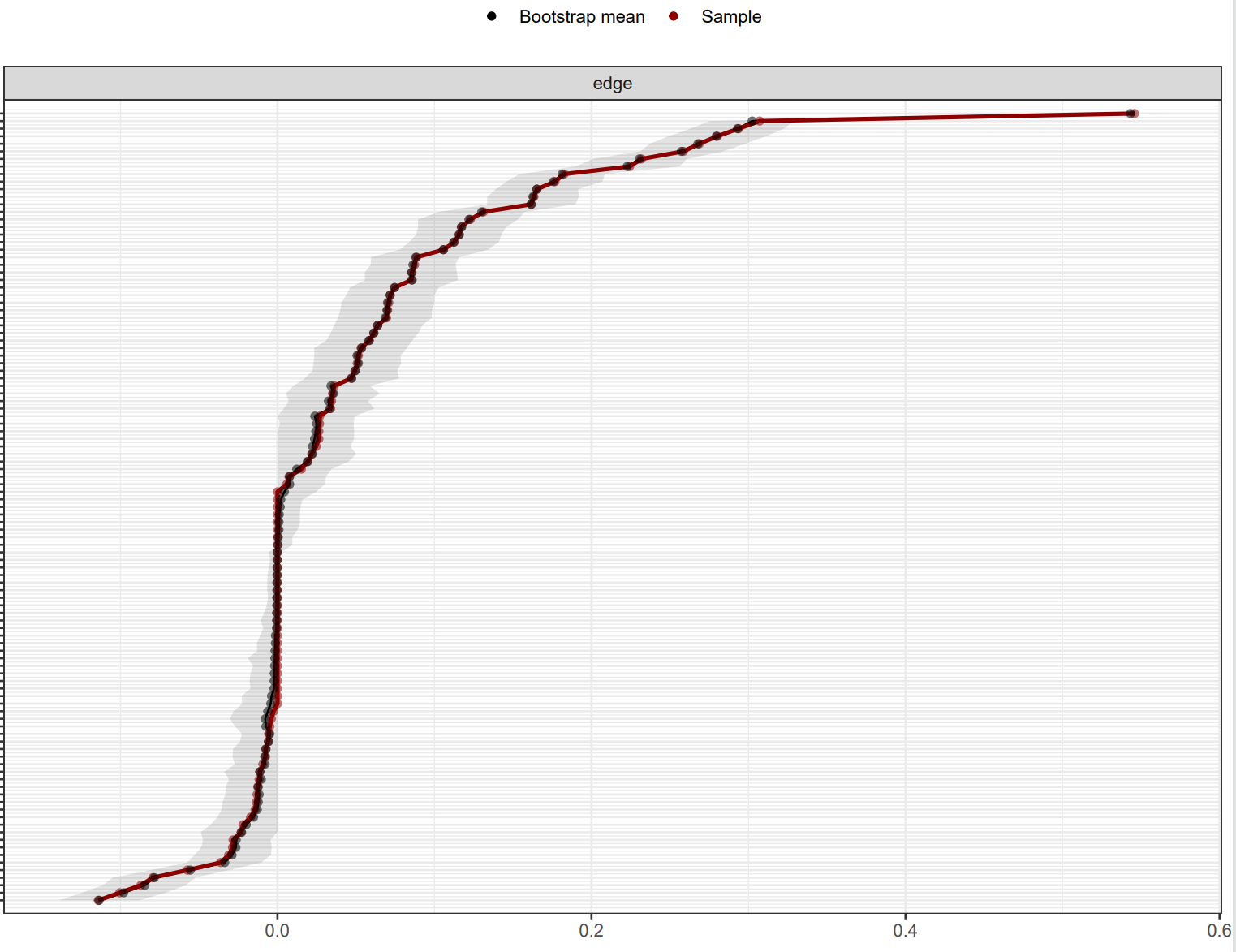


**Wave 2**


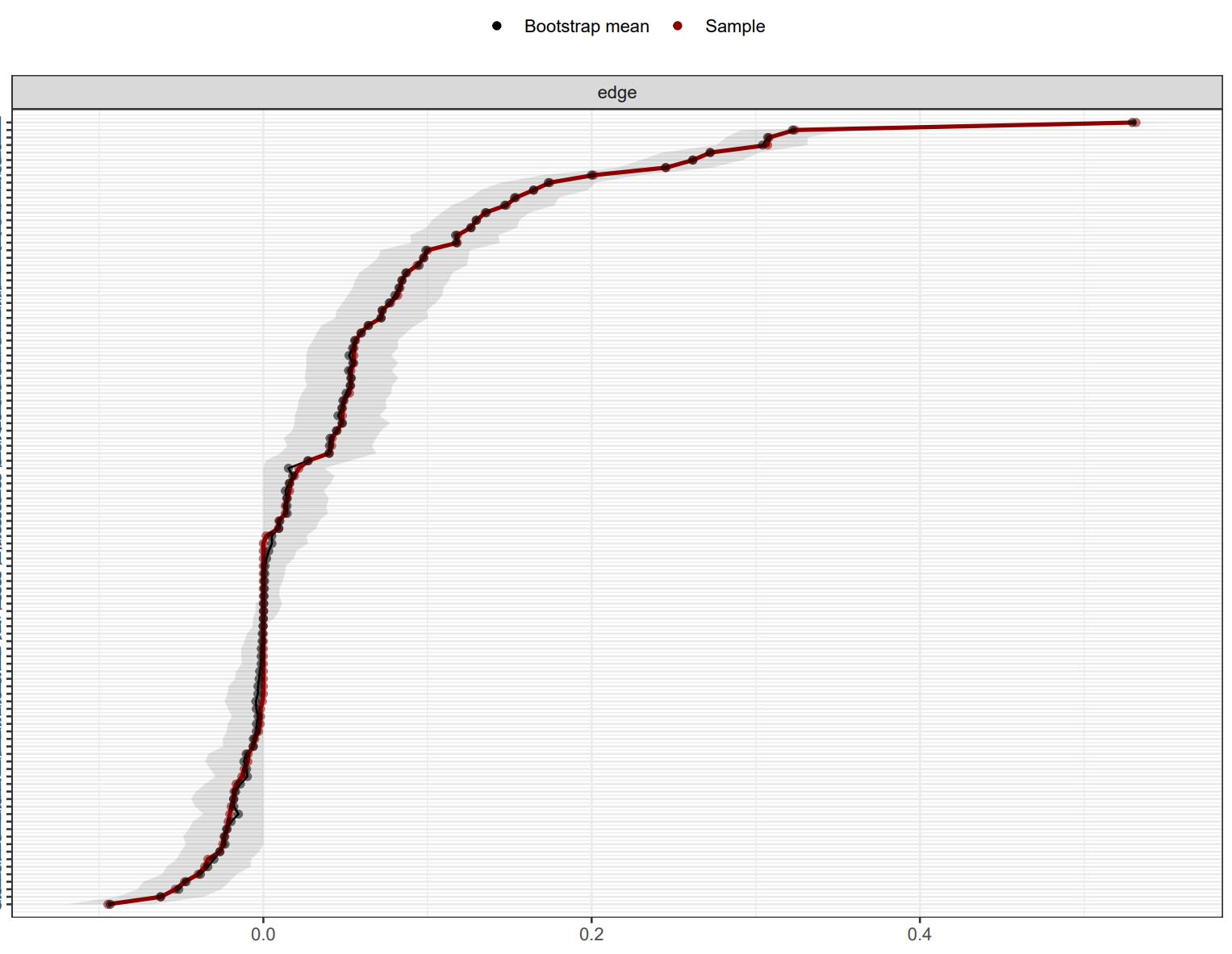


**Wave 3**

Note: The red line indicates the edge weight values, and the gray area indicates the 95% CIs. The wider the bootstrapped CI is for one edge, the more careful the inferences should be.

Fig. S5. Centrality stability in the network using case-drop bootstrapping at three different waves


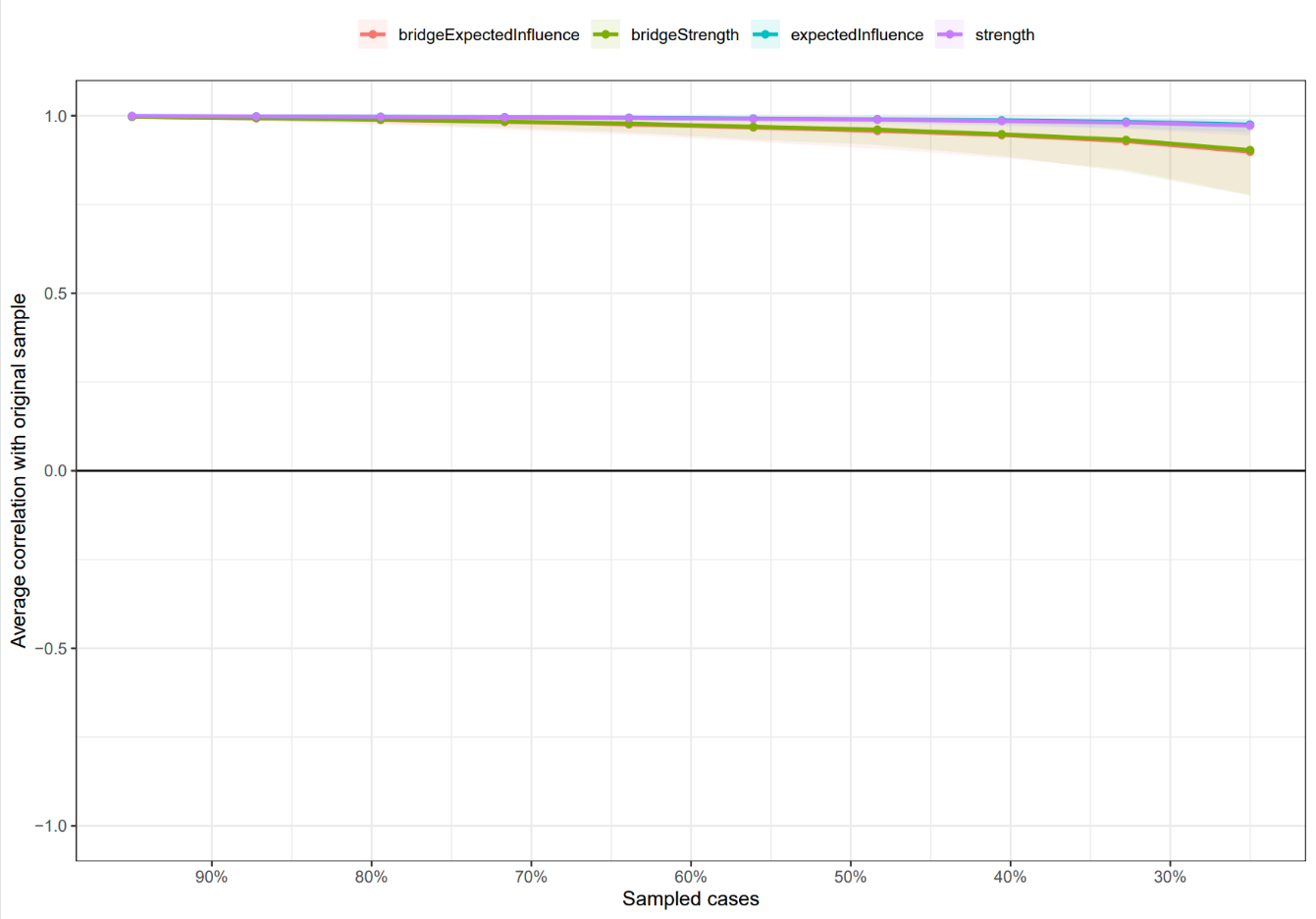


**Wave 1**


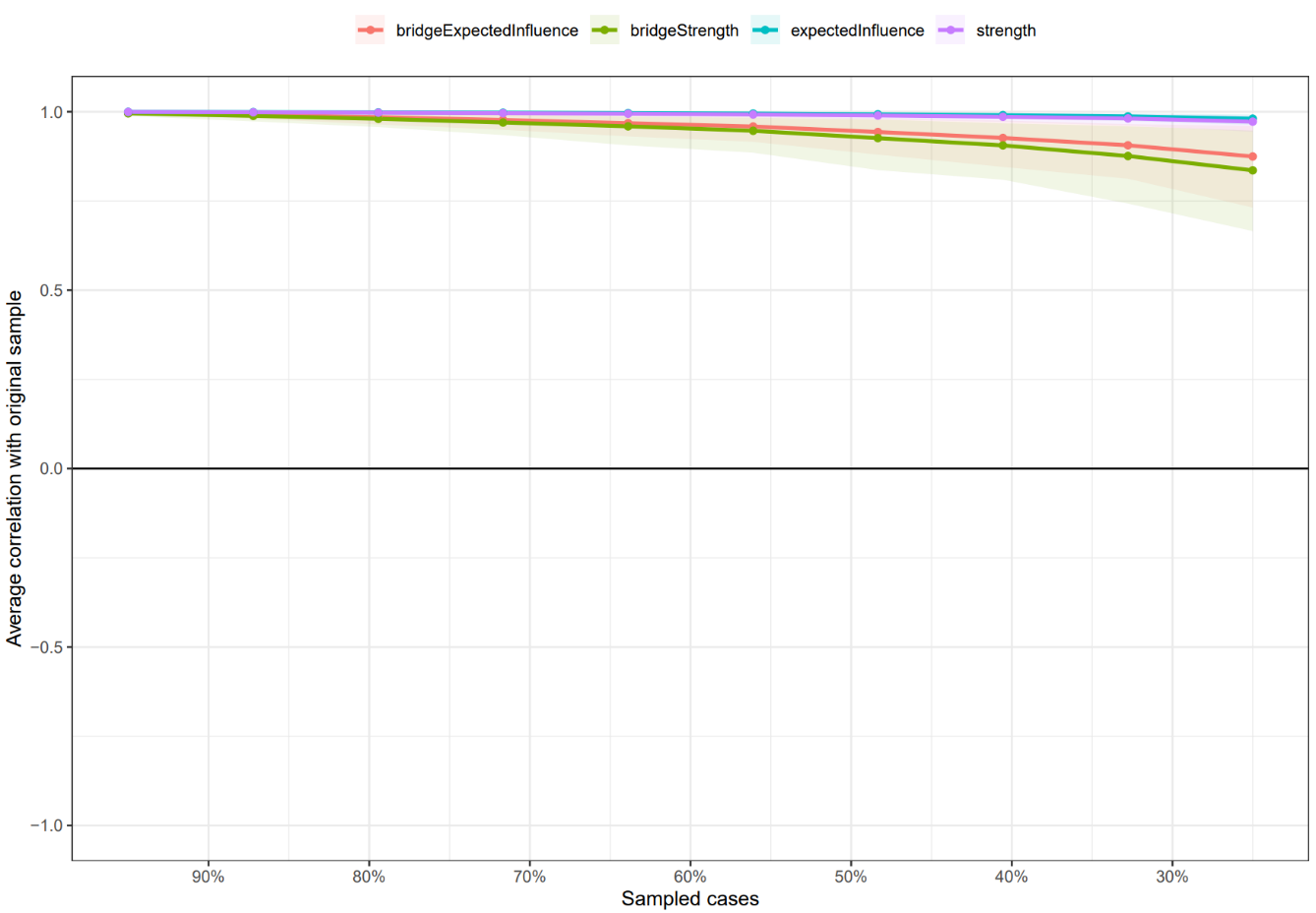


**Wave 2**


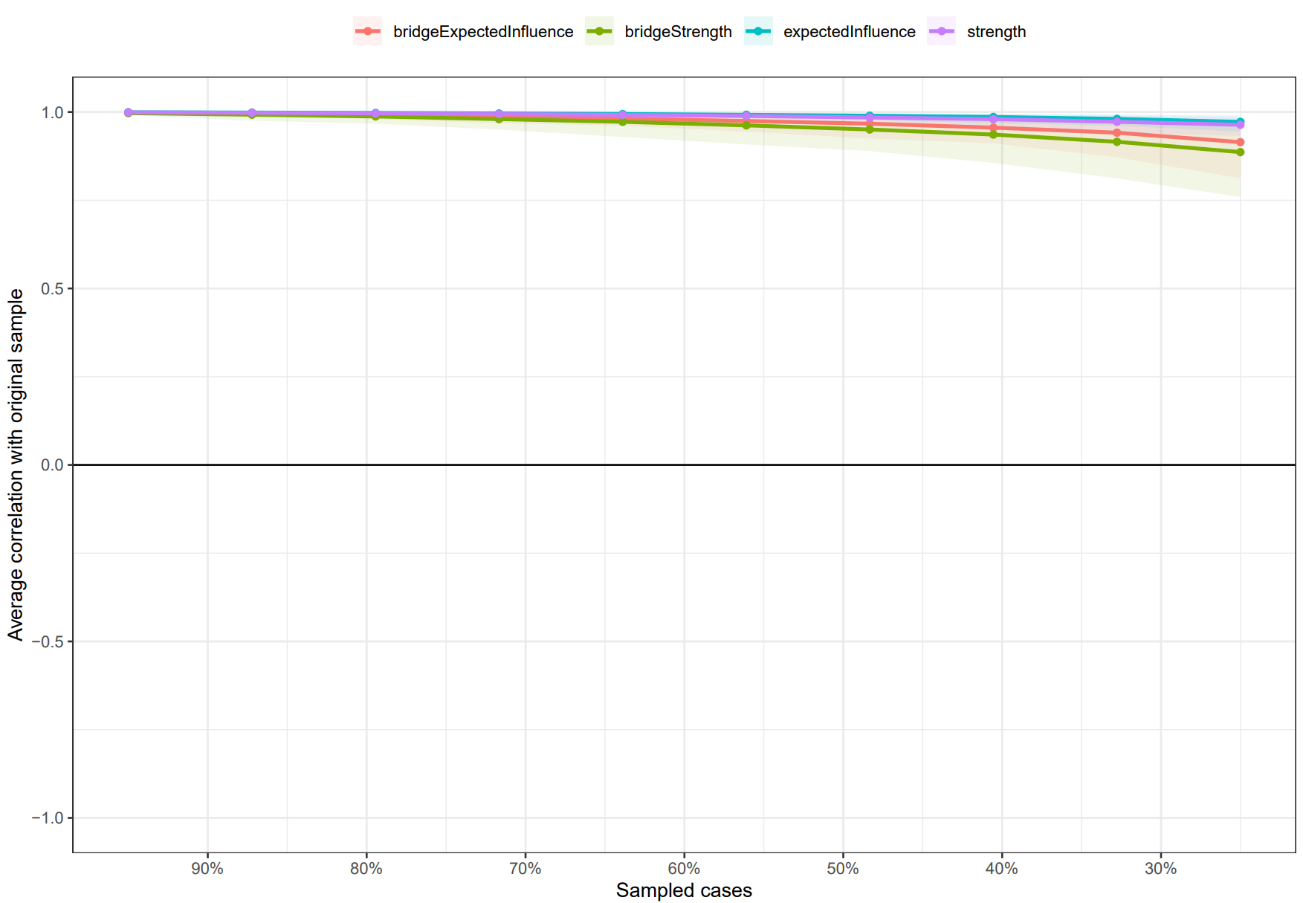


**Wave 3**

Fig. S6. Edge weight difference tests for the network of cognitive function and depression symptoms at three different waves


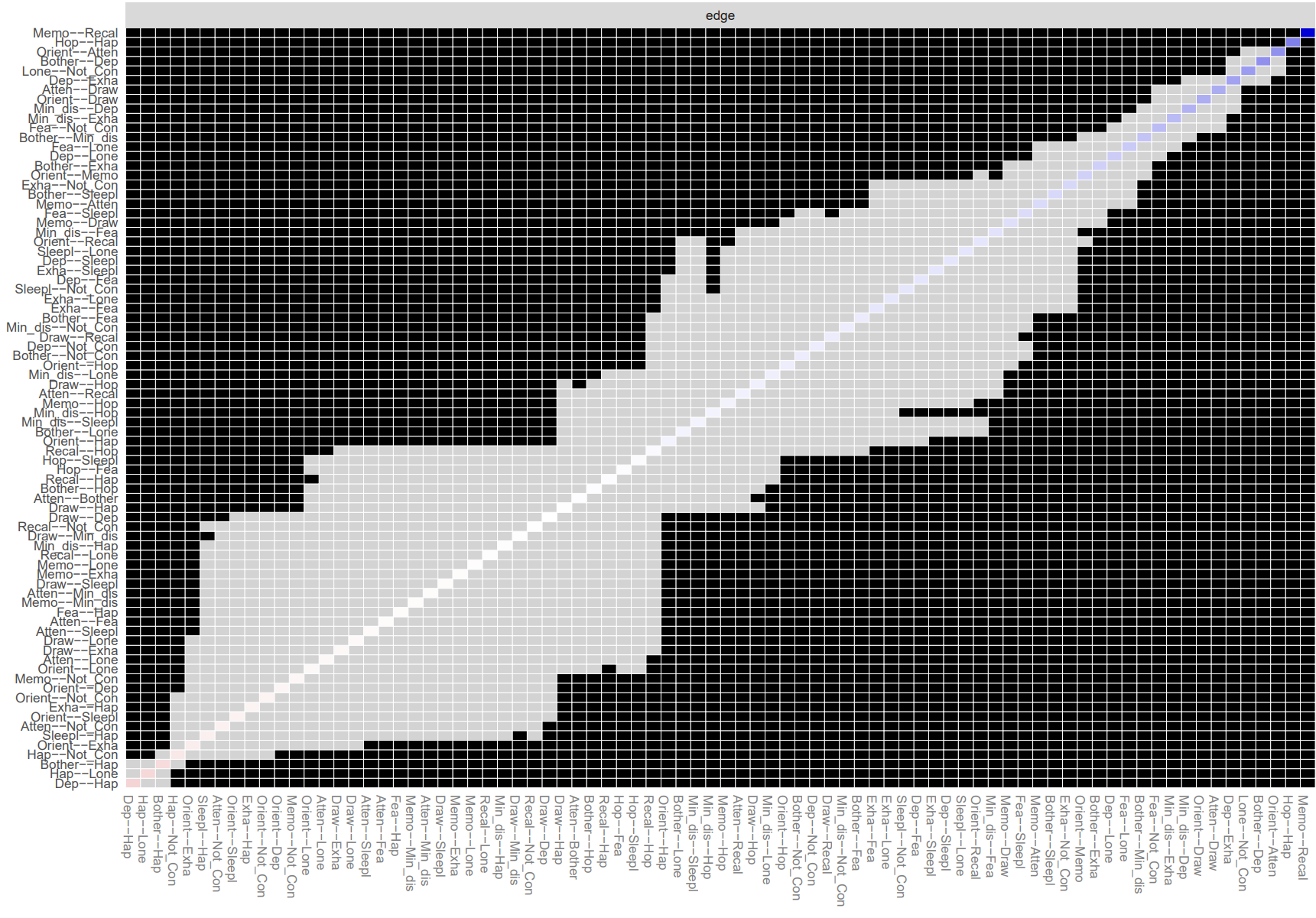


**Wave 1**

**
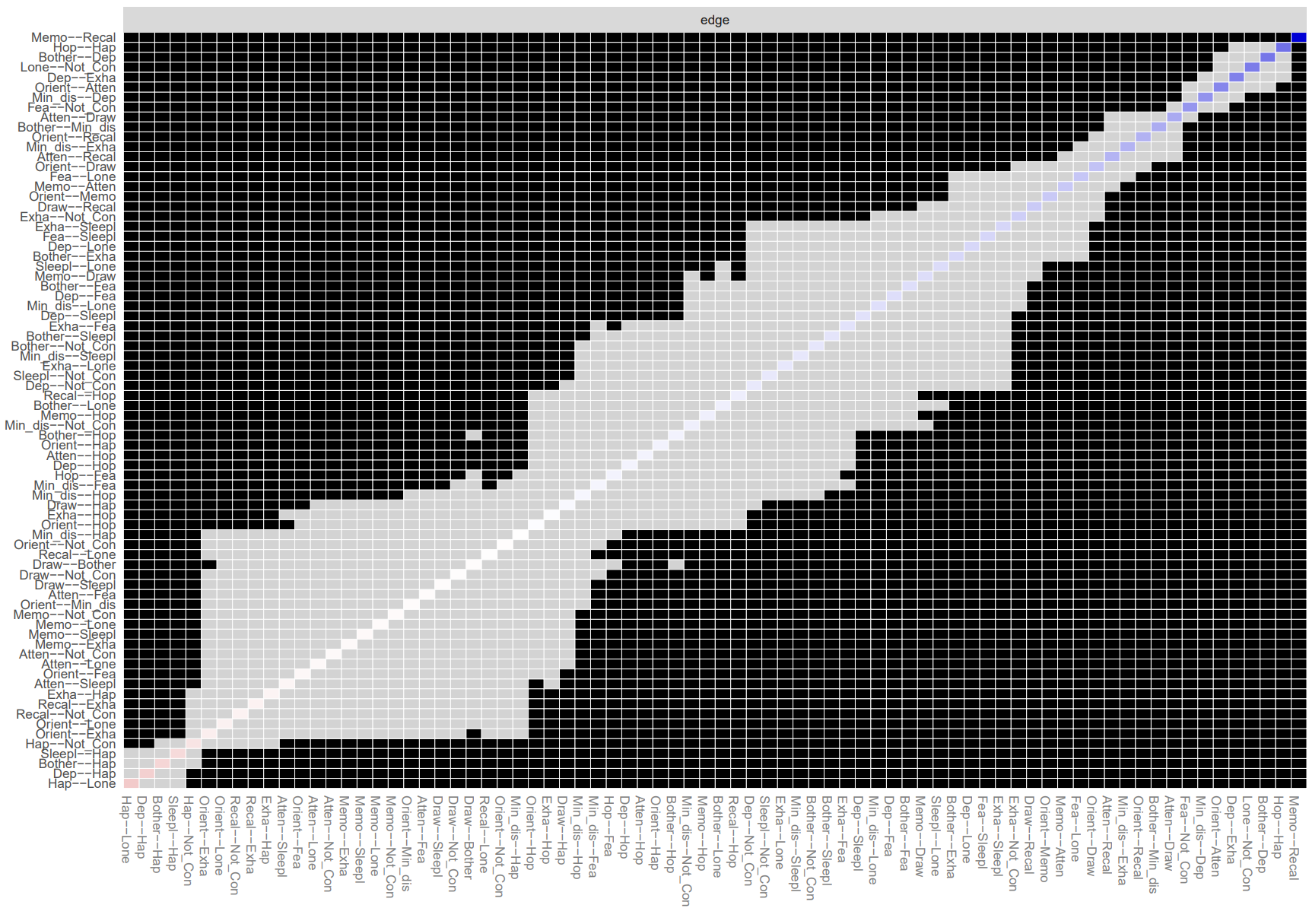
**

**Wave 2**

**
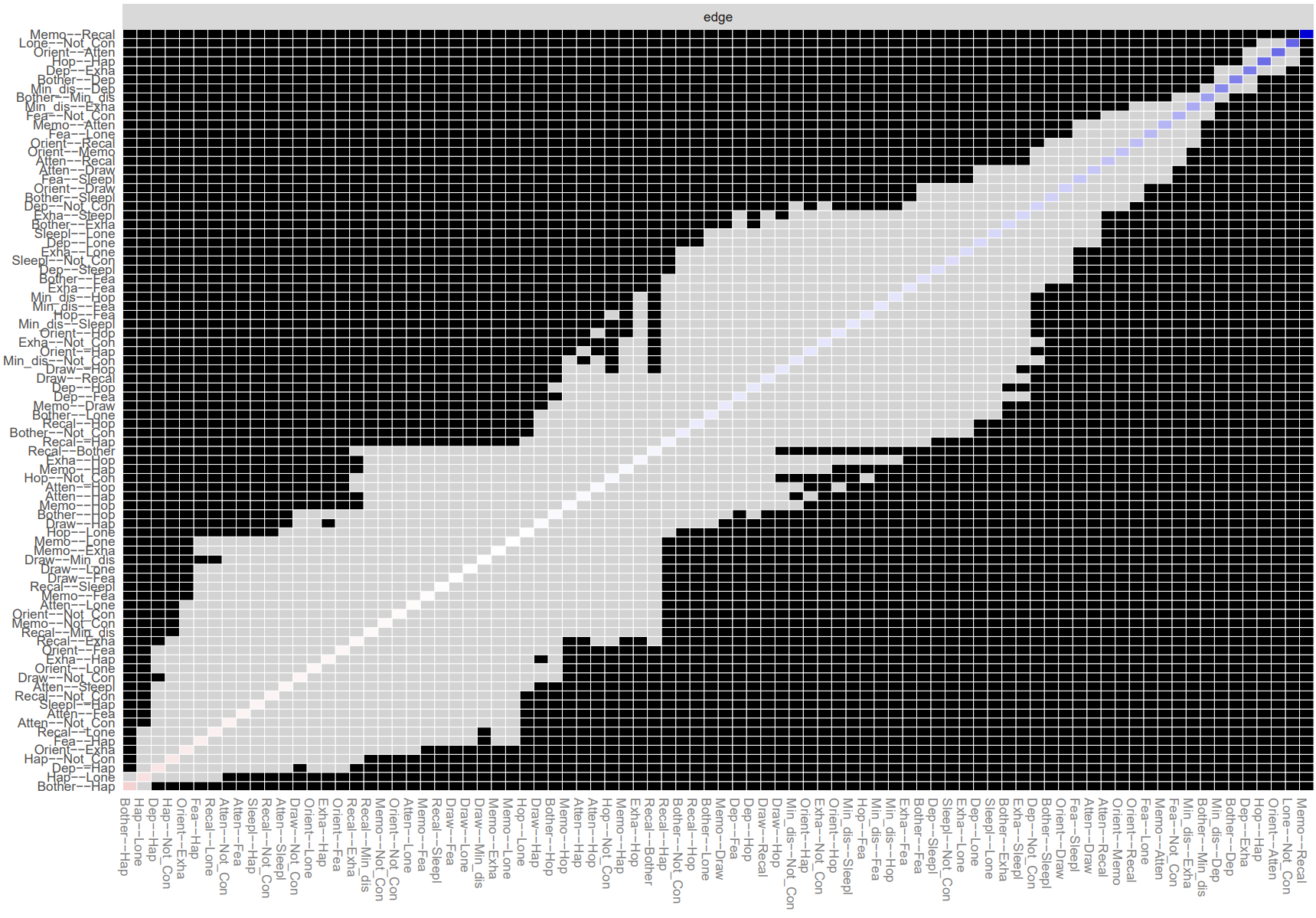
**

**Wave 3**

**
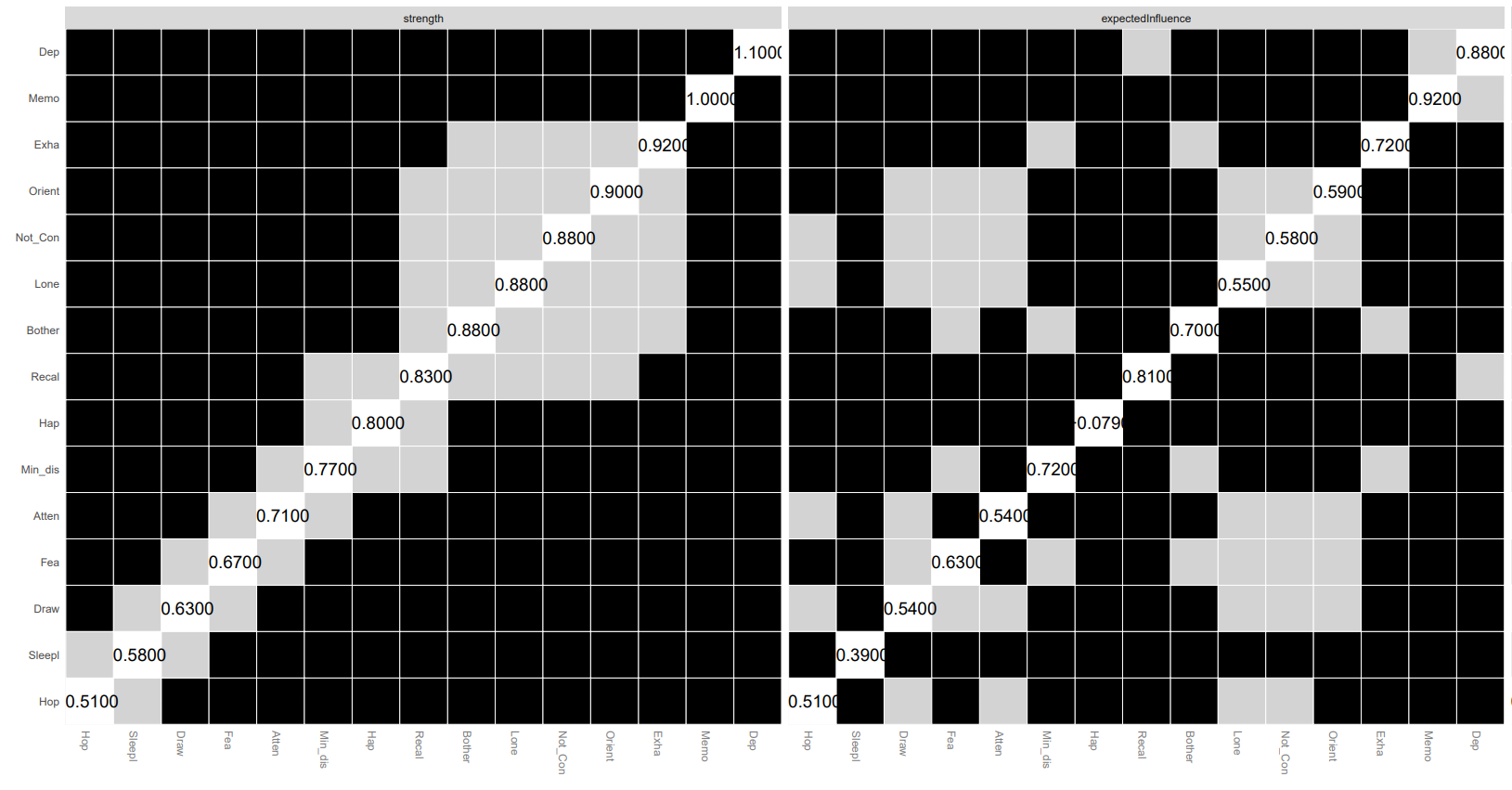
**Fig. S7. Node centrality difference tests for the network of cognitive function and depression symptoms at three different waves


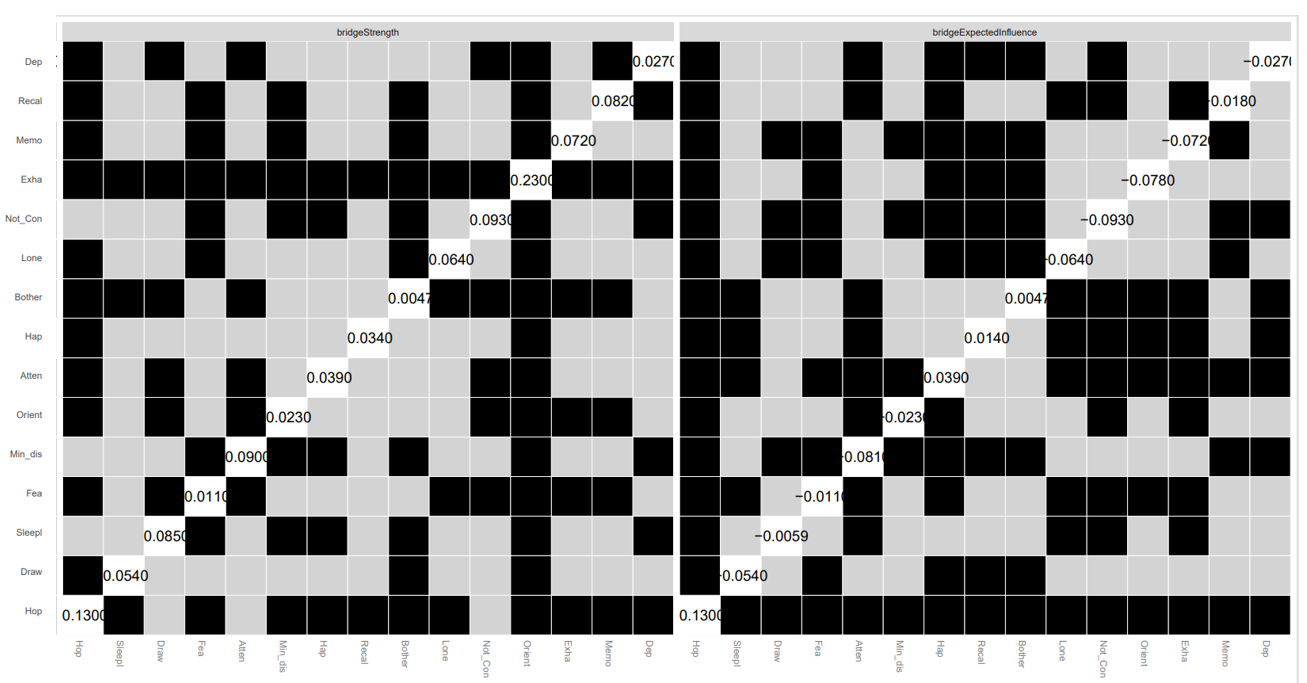


**Wave 1**


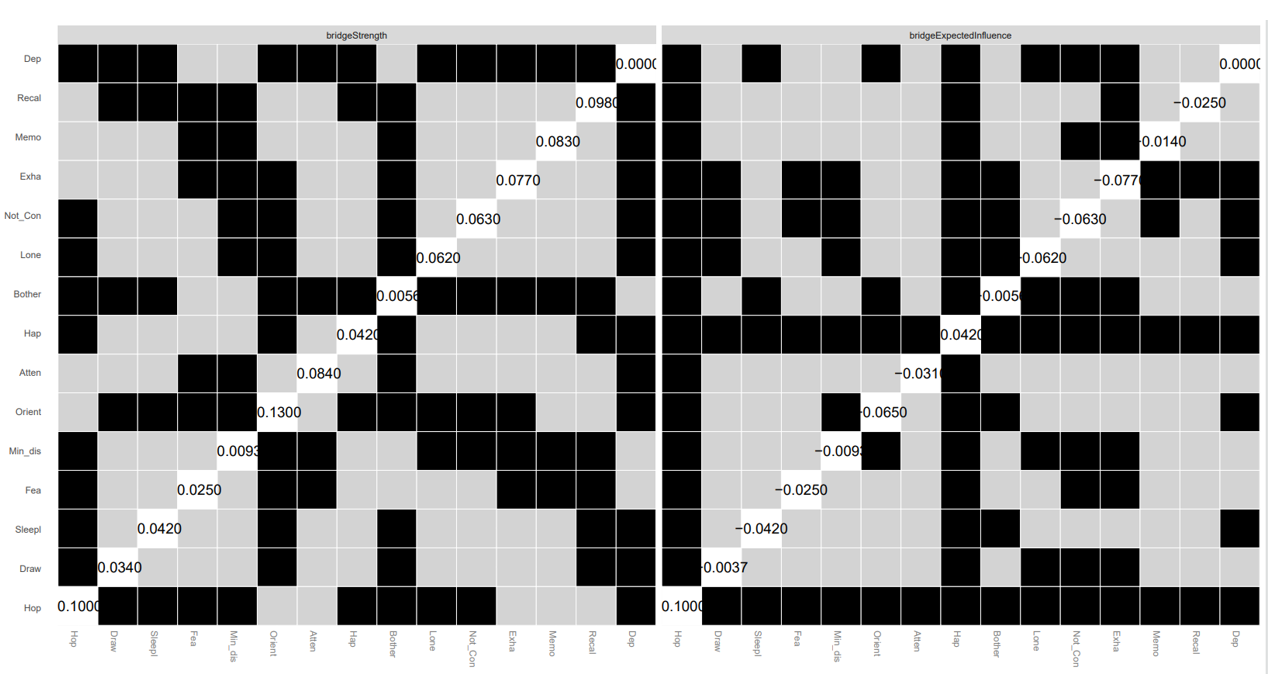

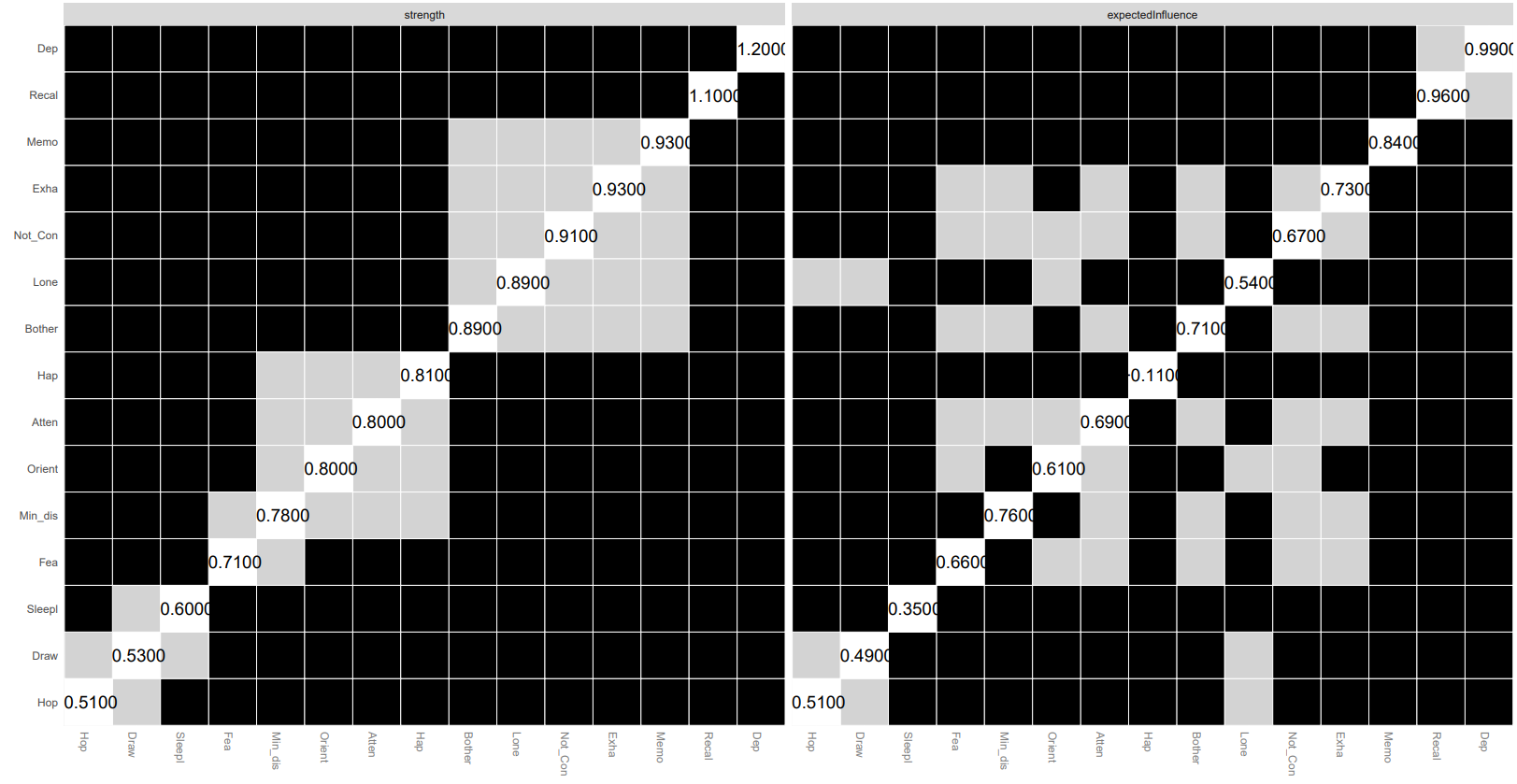
**Wave 2**

**Wave
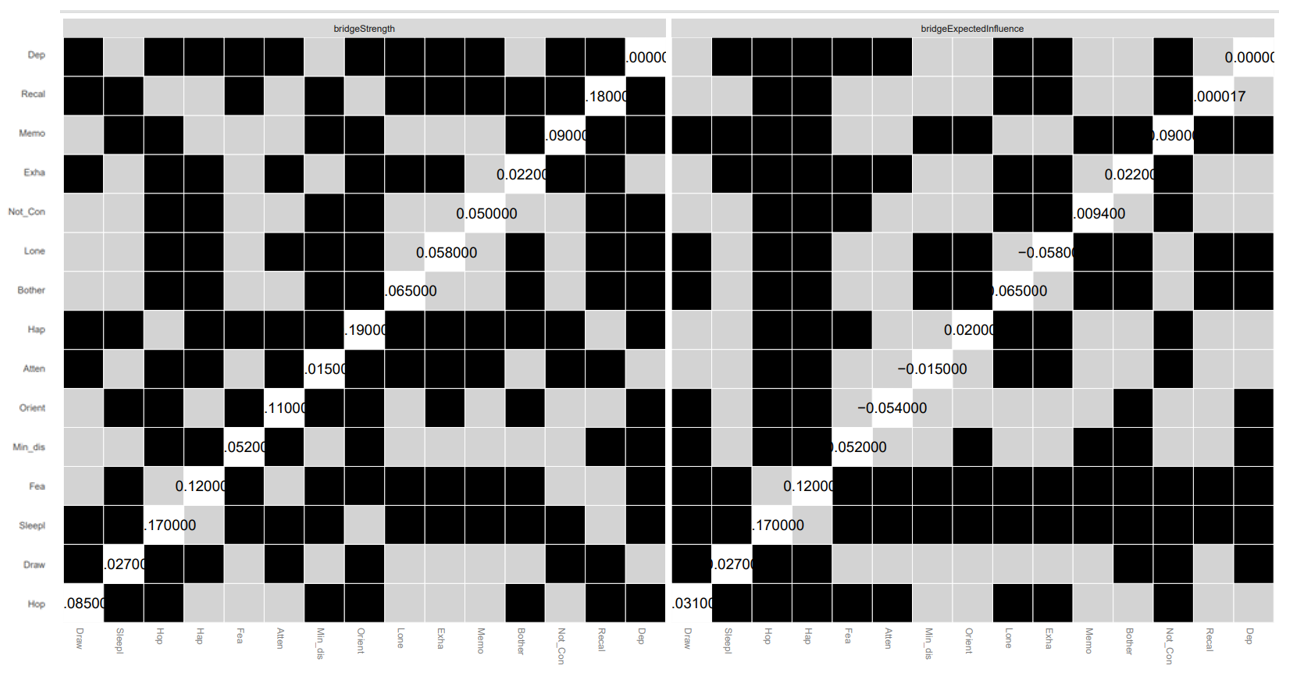
**
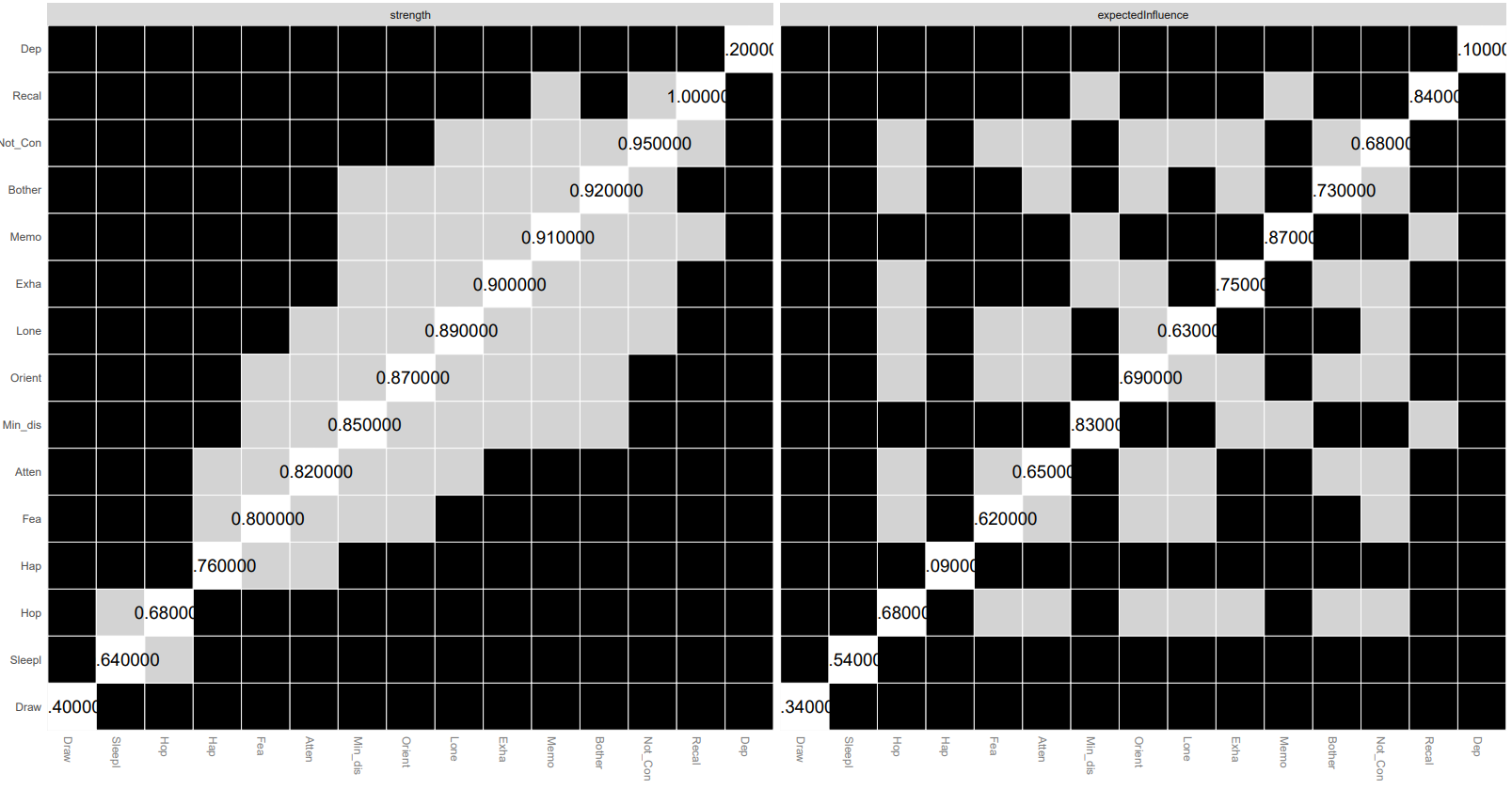
**3**

Fig. S8. The autoregressive impact of network nodes from Wave1 to Wave2 and from Wave2 to Wave3

**
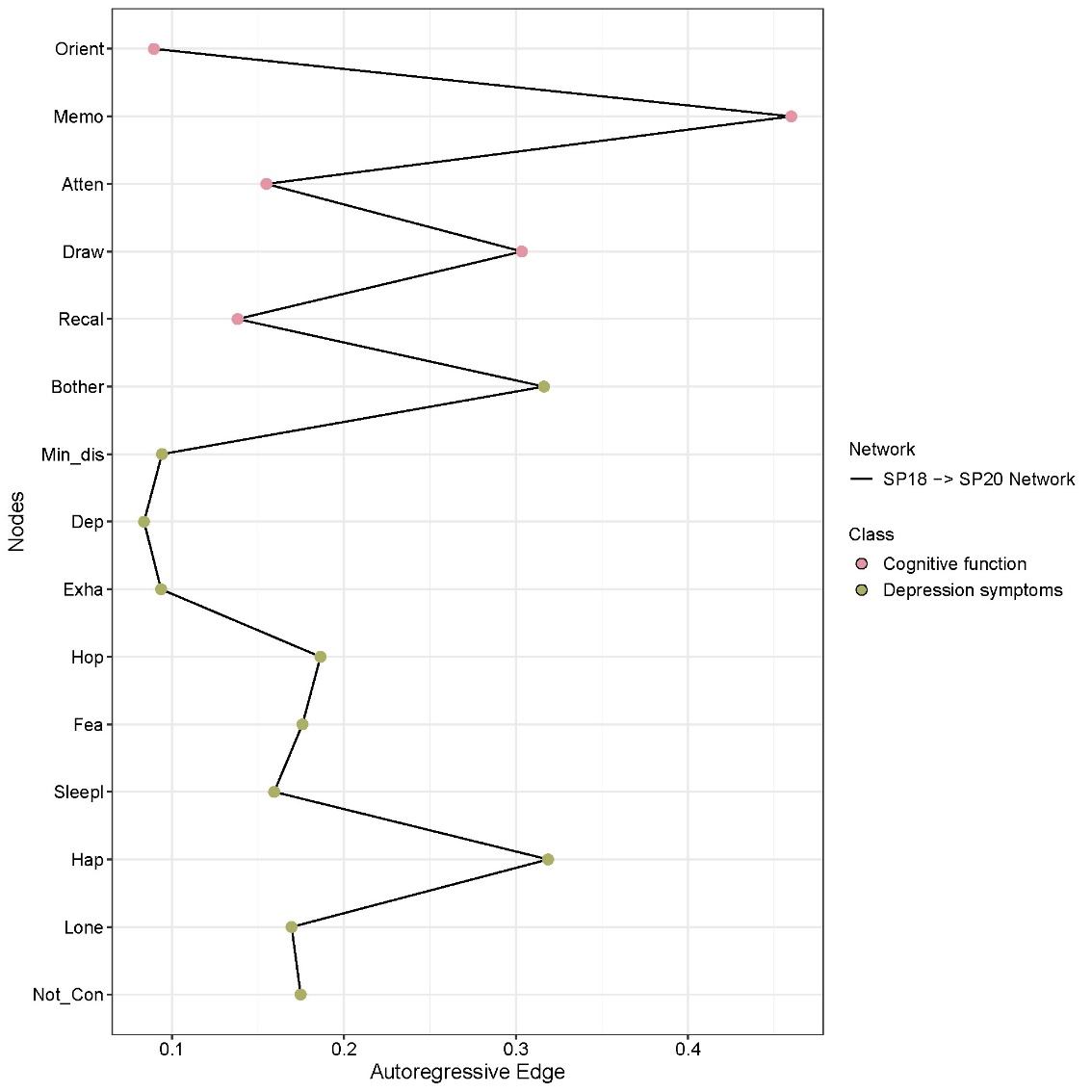

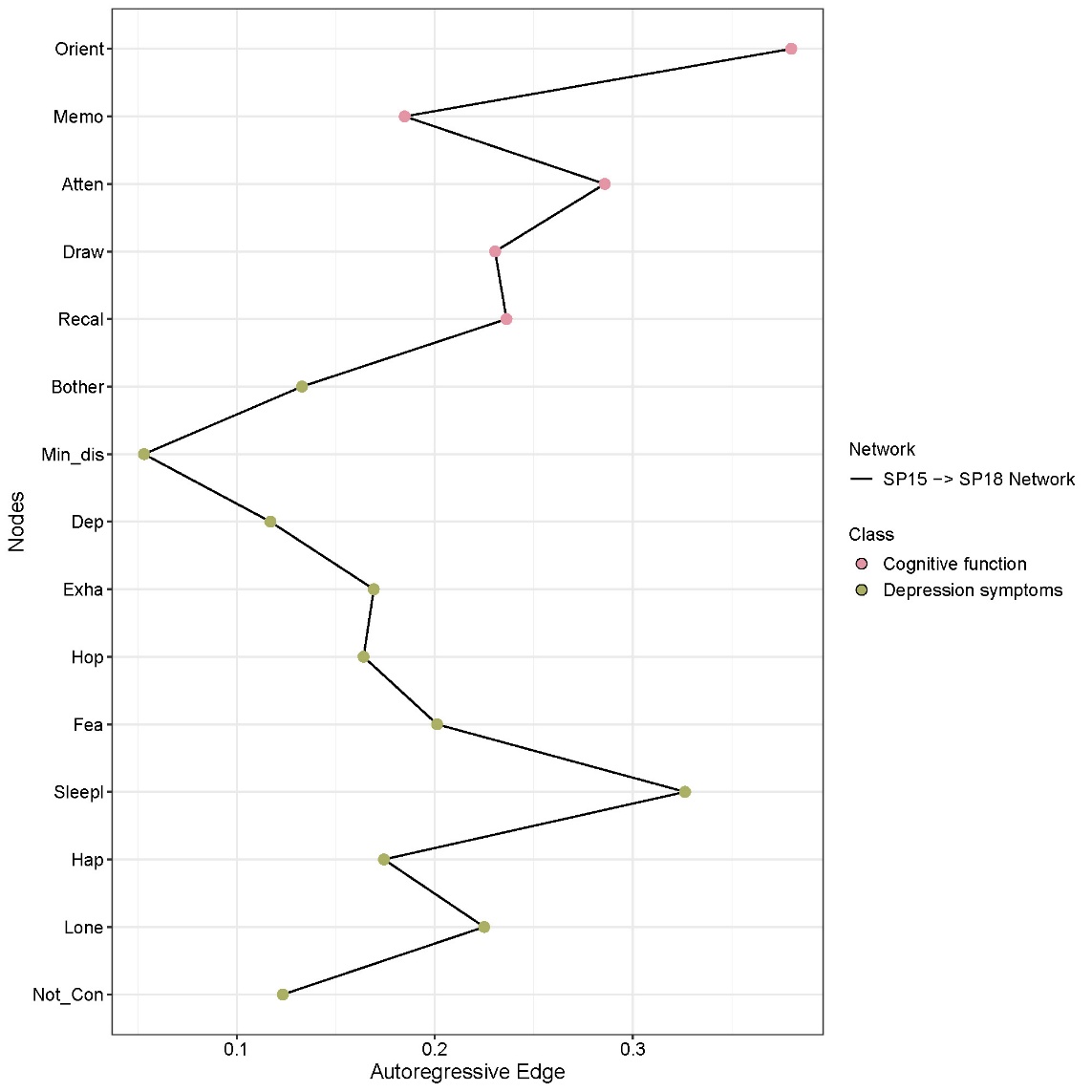
**

Wave1→Wave2 Wave 2→Wave3

Fig. S9. Accuracy of the edge-weight estimates of the temporal network


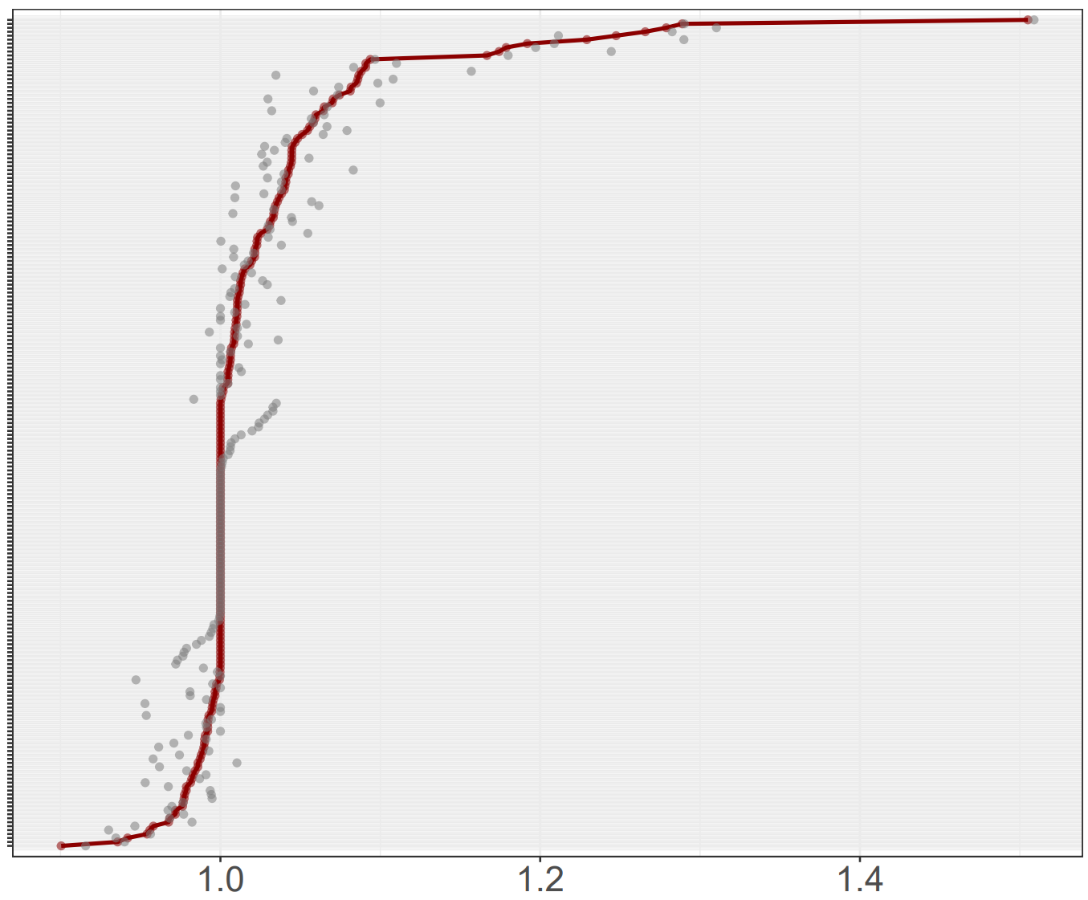


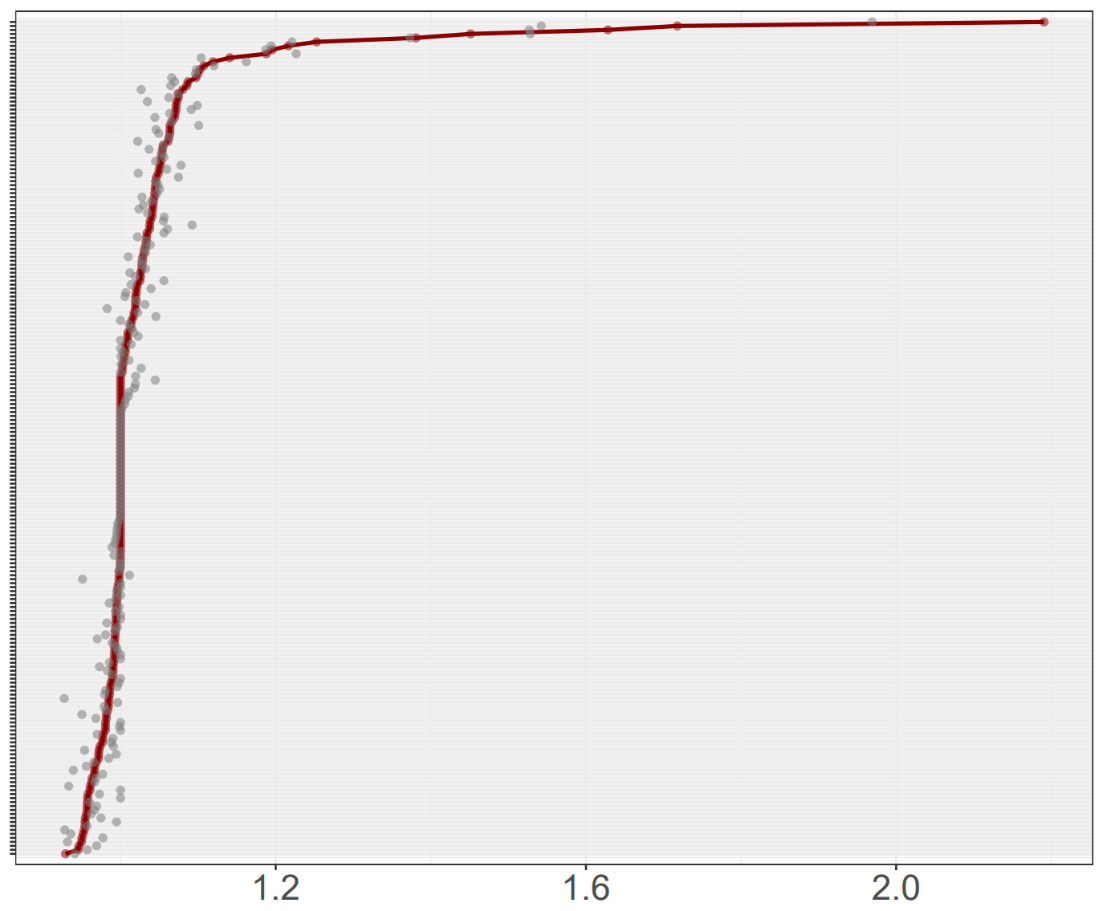
 Wave 1→Wave 2 Wave 2→Wave 3

Fig. S10. Stability of central indices of the temporal network


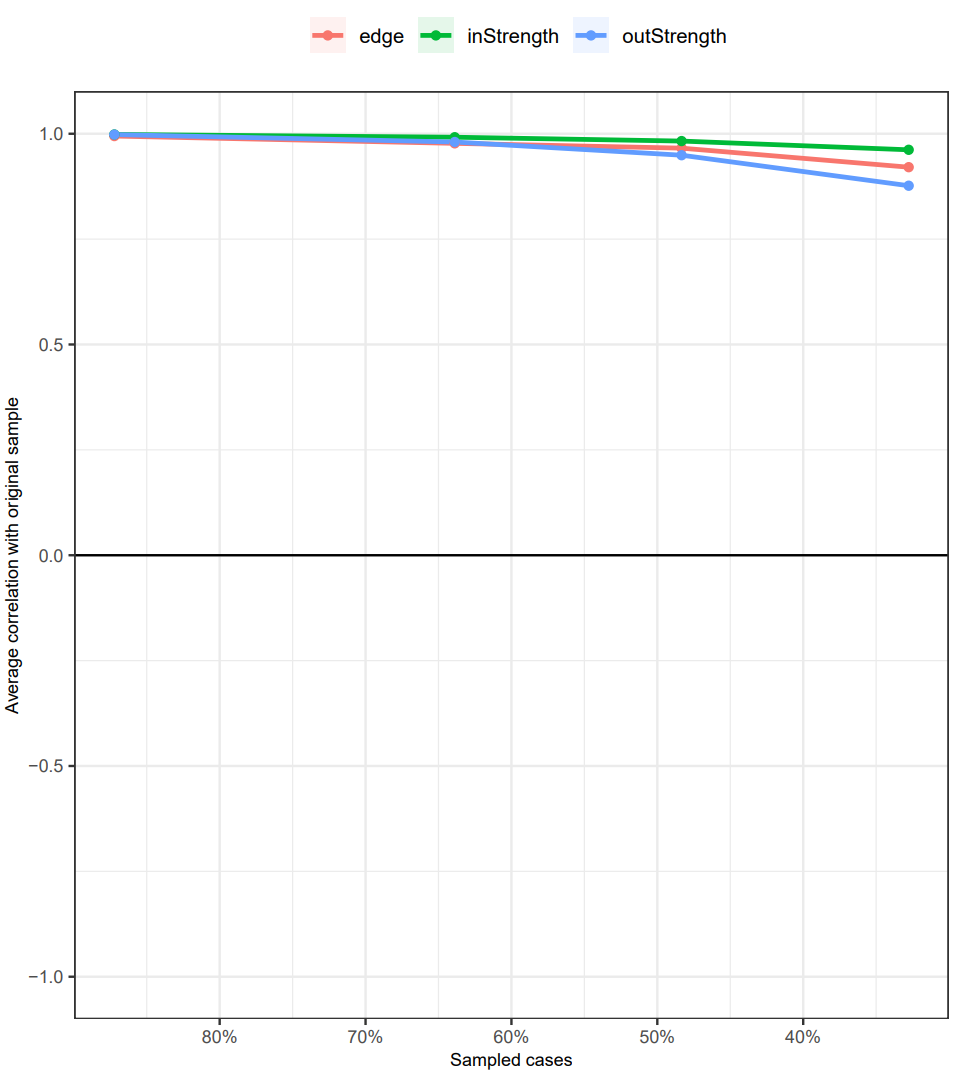

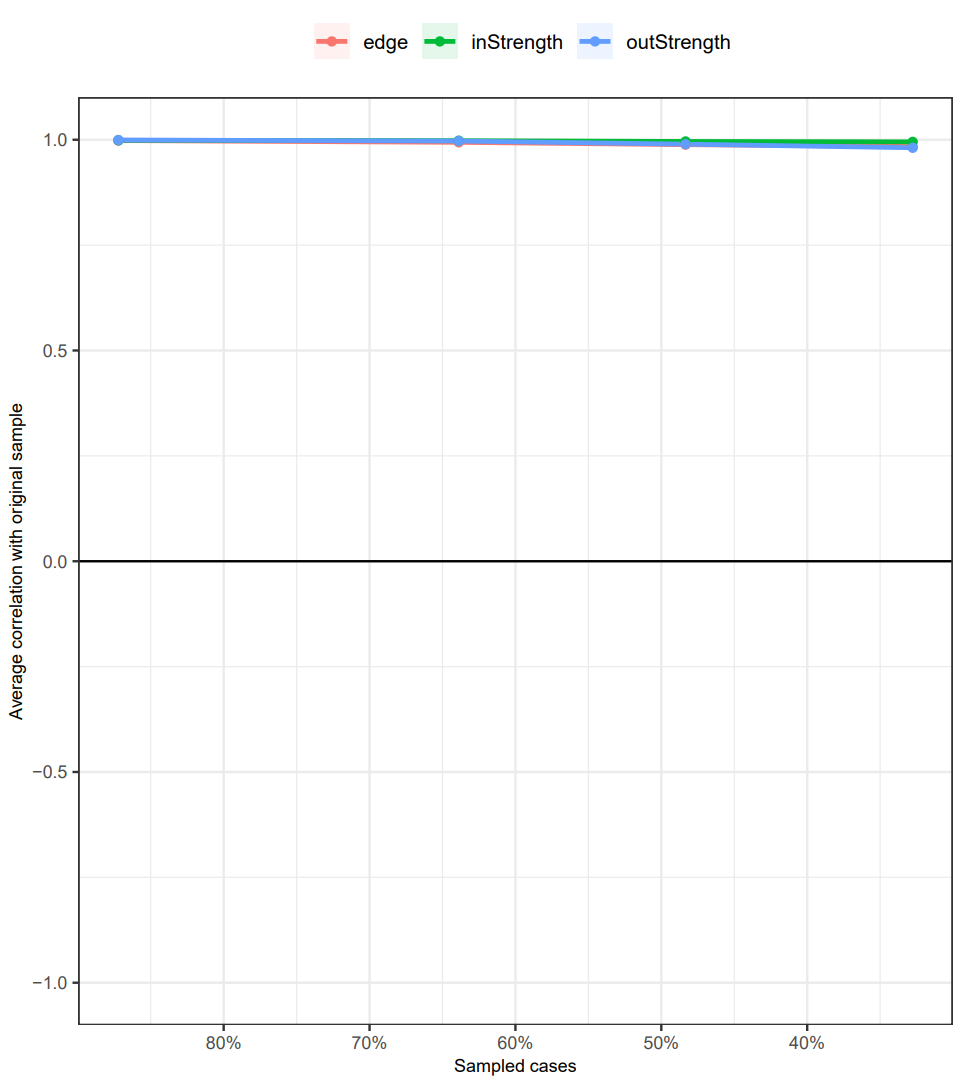


Wave 1→Wave 2 Wave 2→Wave 3
